# Therapeutic knockdown of miR-320 improves deteriorated cardiac function in a pre-clinical model of non-ischemic diabetic heart disease

**DOI:** 10.1016/j.omtn.2022.07.007

**Published:** 2022-07-13

**Authors:** Nilanjan Ghosh, Sonya Fenton, Isabelle van Hout, Gregory T. Jones, Sean Coffey, Michael J.A. Williams, Ramanen Sugunesegran, Dominic Parry, Philip Davis, Daryl O. Schwenke, Anirudha Chatterjee, Rajesh Katare

**Affiliations:** 1 Department of Physiology-HeartOtago, School of Biomedical Sciences, University of Otago, Dunedin, New Zealand; 2 Department of Surgical Sciences, University of Otago, Dunedin, New Zealand; 3 Department of Medicine, University of Otago, Dunedin, New Zealand; 4 Department of Cardiothoracic Surgery, University of Otago, Dunedin, New Zealand; 5 Department of Pathology, Dunedin School of Medicine, University of Otago, Dunedin, New Zealand; 6 Honorary Professor, UPES University, Dehradun, India

**Keywords:** non-ischemic diabetic heart disease, microRNA, miR-320, locked nucleic acid, apoptosis, insulin growth factor-1, microangiopathy, fibrosis

## Abstract

Non-ischemic diabetic heart disease (NiDHD) is characterized by diastolic dysfunction and decreased or preserved systolic function, eventually resulting in heart failure. Accelerated apoptotic cell death because of alteration of molecular signaling pathways due to dysregulation in microRNAs (miRNAs) plays a significant role in the development of NiDHD. Here, we aimed to determine the pathological role of cardiomyocyte-enriched pro-apoptotic miR-320 in the development of NiDHD. We identified a marked upregulation of miR-320 that was associated with downregulation of its target protein insulin growth factor-1 (IGF-1) in human right atrial appendage tissue in the late stages of cardiomyopathy in type 2 diabetic db/db mice and high-glucose-cultured human ventricular cardiomyocytes (AC-16 cells). *In vitro* knockdown of miR-320 in high-glucose-exposed AC-16 cells using locked nucleic acid (LNA) anti-miR-320 markedly reduced high-glucose-induced apoptosis by restoring IGF-1 and Bcl-2. Finally, *in vivo* knockdown of miR-320 in 24-week-old type 2 diabetic db/db mice reduced cardiomyocyte apoptosis and interstitial fibrosis while restoring vascular density. This resulted in partial recovery of the impaired diastolic and systolic function. Our study provides evidence that miR-320 is a late-responding miRNA that aggravates apoptosis and cardiac dysfunction in the diabetic heart, and that therapeutic knockdown of miR-320 is beneficial in partially restoring the deteriorated cardiac function.

## Graphical abstract

**Figure:**
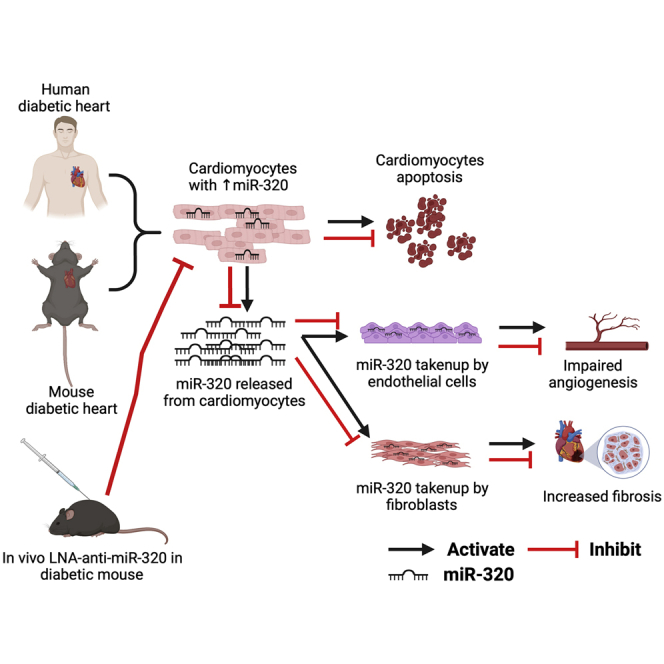

## Teaser

Non-ischemic diabetic heart disease is characterized by cardiac dysfunction and apoptosis. In this study, we identified upregulation of miR-320 as the underlying mechanism. This was supported by restoration of cardiac function and reduced apoptosis after knockdown of miR-320 in a type 2 diabetic mouse model.

## Introduction

More than 70% of individuals with diabetes develop some form of heart disease during their life span.^1^ Notably, the risk of heart failure increases 2- to 3-fold in the presence of diabetes.^2^^,^^3^ The impact of diabetes on vascular and ischemic disease, such as coronary and cerebrovascular disease, is well established, but non-ischemic cardiac complications associated with diabetes have received relatively little attention. Non-ischemic diabetic heart disease (NiDHD) is a chronic complication characterized by ventricular dilation and hypertrophy, diastolic dysfunction, decreased or preserved systolic function, eventually resulting in heart failure.^4^^,^^5^ While numerous factors have been associated with the development of NiDHD,^6^, ^7^, ^8^, ^9^ the fundamental mechanisms leading to NiDHD are still not known. Abnormal levels of glycated hemoglobin have been linked to the development of NiDHD. However, studies have demonstrated that intensive glycemic control was insufficient in reducing the risk of heart failure among patients with diabetes mellitus.^10^ Accumulating evidence suggests that alteration in the molecular signaling pathways due to dysregulation in microRNAs (miRNAs) plays a crucial role in developing NiDHD.^11^^,^^12^

miRNAs are small non-coding RNAs that have well-established functions in cardiovascular diseases, cancer, and metabolic disorders and are described as the “micromanagers” of gene expression.^13^ We, along with others, have identified the crucial role of miRNAs in the diabetic heart.^14^, ^15^, ^16^, ^17^, ^18^, ^19^ We demonstrated that dysregulation in miRNAs is associated with vascular rarefaction, increased fibrosis, and accelerated aging in the diabetic heart.

Cardiomyocytes are highly susceptible to apoptosis in diabetic conditions.^20^ Previous studies showed that miR-320 is expressed abundantly in cardiomyocytes^21^^,^^22^ and directly targets the pro-survival and anti-apoptotic gene insulin growth factor-1 (IGF-1).^22^^,^^23^ IGF-1 is an important regulator that maintains myocardial structural integrity and homeostasis.^21^ Overexpression of miR-320 in cardiomyocytes downregulated the expression of anti-apoptotic protein Bcl-2 while increasing the expression of pro-apoptotic proteins Bax and caspase-3, the downstream signaling cascade of IGF-1, leading to increased cell death, suggesting a pathological role for miR-320 in the heart.^24^

In this study, we demonstrate the upregulation of pro-apoptotic miR-320 in the heart of individuals with type 2 diabetes, type 2 diabetic (db/db) mice, and in high glucose (HG)-cultured human ventricular cardiomyocytes (AC-16 cells). This was associated with the downregulation of pro-survival IGF-1, the direct target of miR-320, resulting in inhibition of its downstream anti-apoptotic signaling cascade. Importantly, *in vitro* and *in vivo* knockdown of miR-320 using locked nucleic acid (LNA) could reverse apoptotic cell death in HG-cultured AC-16 cells and db/db mice, rescuing the impaired cardiac function.

## Results

The uncropped western blots and full histological images are presented as figures in the supplemental information.

### Diabetes upregulates miR-320 in the human heart

**Figure 1:**
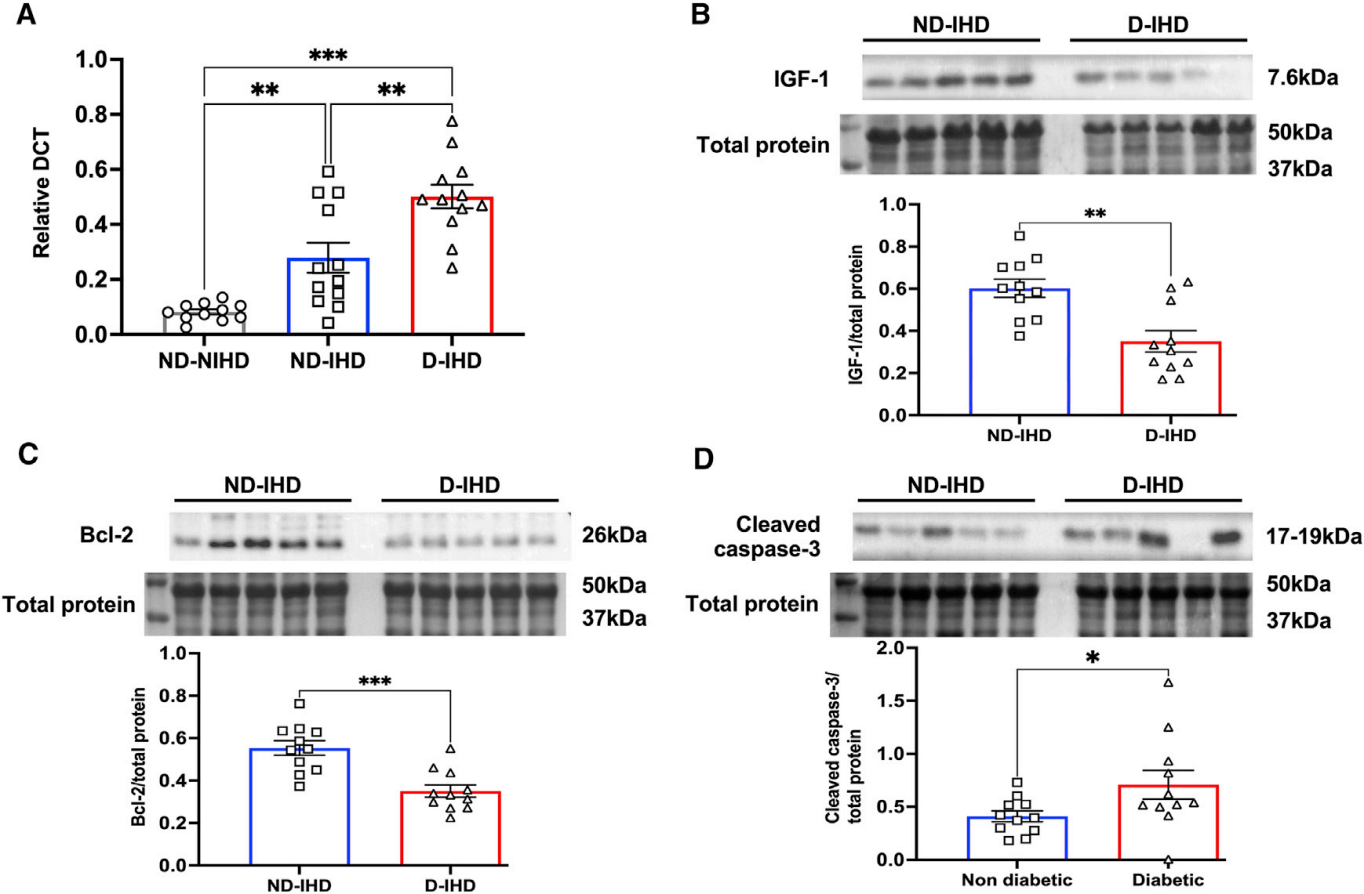
Diabetes upregulates miR-320 in the human heart (A) Quantitative scatterplot bar graph showing miR-320 expression in the RAA tissue by RT-PCR analysis. Samples were collected from diabetic (D) and non-diabetic (ND) patients with ischemic heart disease (IHD) undergoing coronary artery bypass graft surgery. Data are mean ± SEM and expressed as relative DCT. (B–D) Representative western blots and quantitative scatterplot bar graphs showing the expression of IGF-1 (B), Bcl-2 (C), and cleaved caspase-3 (D) in the study groups. Data are represented as the ratio to total protein and are mean ± SEM. Each western blot analysis was repeated three independent times. ∗p < 0.05, ∗∗p < 0.01, and ∗∗∗p < 0.001.

RT-PCR analysis showed significant upregulation of miR-320 in right atrial appendage (RAA) biopsies collected from non-diabetic (ND) patients with ischemic heart disease (IHD) compared with the hearts collected from ND cadavers without any history of diabetes or IHD (ND-NIHD) (p = 0.006; Figure 1A). However, diabetes-induced further upregulation of miR-320 (p = 0.001 versus ND-IHD; Figure 1A). Importantly, this was associated with marked downregulation of IGF-1 (p = 0.0024, versus ND-IHD; Figures 1B and S1A). IGF-1 is a pro-survival protein that regulates anti-apoptotic activity by regulating Bcl-2 and caspase activity. In line with this, western blot analysis showed significant downregulation of Bcl-2 (p = 0.0006 versus ND-IHD; Figures 1C and S1B), while apoptotic marker cleaved caspase-3 was upregulated (p = 0.040 versus ND-IHD; Figures 1D and S1D).

### miR-320 upregulates in the later stage of diabetes

**Figure 2:**
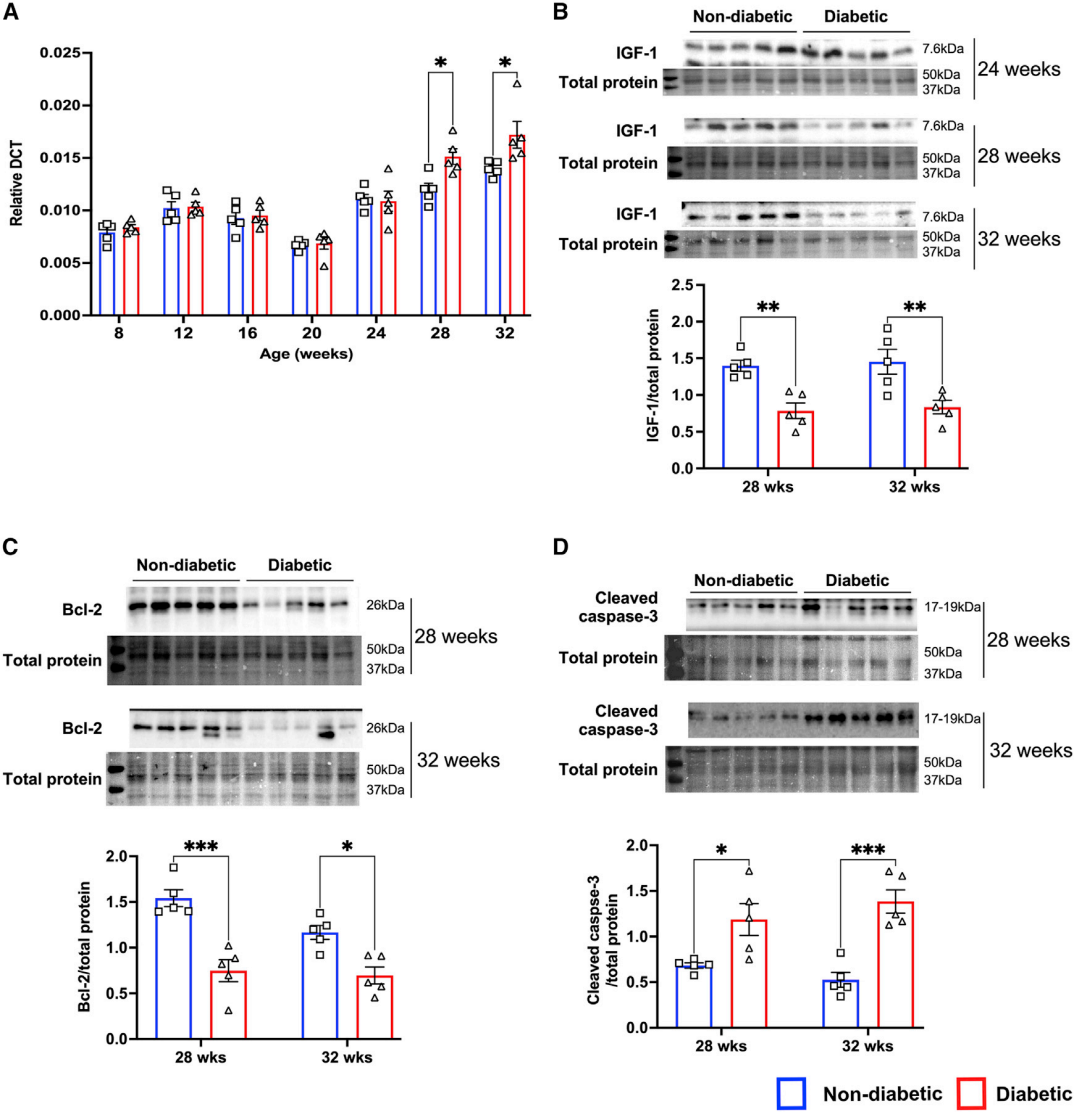
miR-320 upregulates in the later stage of diabetes (A) Quantitative scatterplot bar graph showing miR-320 expression in the heart by RT-PCR analysis. Samples were collected from obese diabetic db/db mice and lean non-diabetic db/+ mice at different time points. Data are mean ± SEM and expressed as relative DCT. (B–D) Representative western blots and quantitative scatterplot bar graphs showing the expression of IGF-1 (B), Bcl-2 (C), and cleaved caspase-3 (D) at different time points in the study groups. Data are represented as the ratio to total protein and are mean ± SEM. Each western blot analysis was repeated three independent times. Bcl-2 and cleaved caspase-3 were tested only at 28 and 32 weeks as the changes in miR-320 and the upstream target IGF-1 was not observed until 28 weeks of age. Other time points for IGF-1 are shown in Figure S2A. ∗p < 0.05 and ∗∗∗p < 0.001. The same image was used in (B) and (C) for total proteins as the membrane used to probe IGF-1 (B) and Bcl-2 (C).

While the RT-PCR analysis confirmed that diabetes induces upregulation of miR-320 in the human heart (Figure 1A), it was not clear whether the upregulation of miR-320 is a causative factor for increased apoptosis in the diabetic heart and whether diabetes alone in the absence of ischemia is able to induce the upregulation of miR-320 that is sufficient to induce apoptosis. Therefore, to establish the mechanistic role for miR-320 in NiDHD, myocardial tissue samples were collected from 8- to 32-week-old db/db mice and their age-matched ND db/+ mice to determine the expression of miR-320 and IGF-1. RT-PCR analysis demonstrated a significant increase in miR-320 at both 28 (p = 0.042) and 32 weeks (p = 0.033) of age in db/db mice (Figure 2A). This was associated with downregulation of target protein IGF-1 at both 28 (p = 0.0138) and 32 (p = 0.0124) weeks (Figures 2B and S2A–S2H). No significant changes were observed in miR-320 and IGF-1 expression from 8 to 24 weeks (Figures 2A and 2B). Western blot analysis further confirmed significant downregulation of anti-apoptotic Bcl-2 (p = 0.0001 at 28 weeks and p = 0.0156 at 32 weeks, Figures 2C, S2I, and S2J) and upregulation of cleaved caspase-3 (p = 0.0340 at 28 weeks and p = 0.0005 at 32 weeks; Figures 2D, S2K, and S2L) in db/db mice.

### HG induces upregulation of miR-320 in cultured human cardiomyocytes

**Figure 3:**
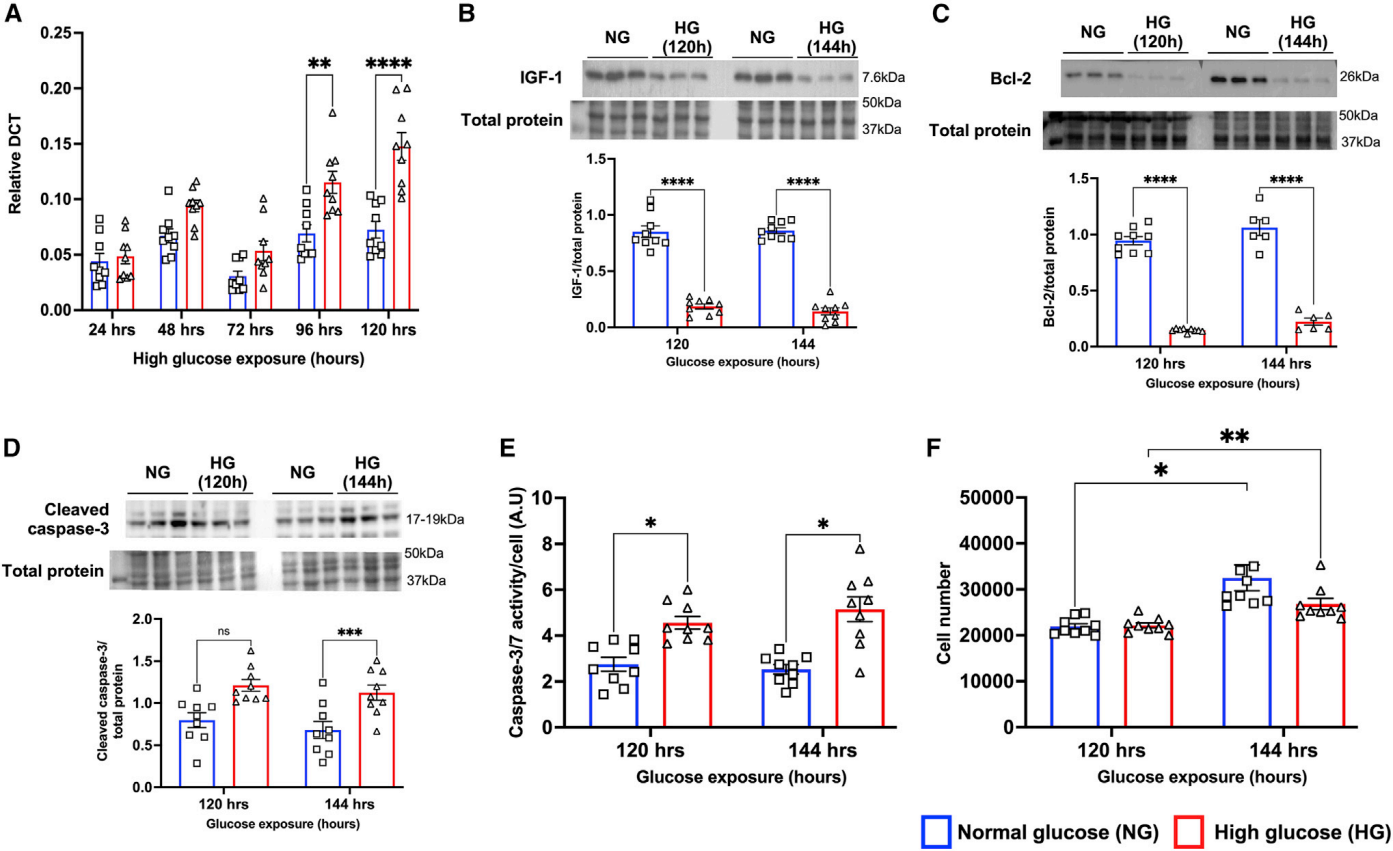
High glucose induces upregulation of miR-320 in cultured human cardiomyocytes (A) Quantitative scatterplot bar graph showing miR-320 expression in the normal glucose (NG) and high glucose (HG)-treated AC-16 human cardiomyocytes by RT-PCR analysis. Samples were collected at different points after exposing the cells to HG or mannitol for osmotic control (NG). Data are mean ± SEM and expressed as relative DCT. (B–D) Representative western blots and quantitative scatterplot bar graphs showing the expression of IGF-1 (B), Bcl-2 (C), and cleaved caspase-3 (D) at different time points in the study groups. Data are represented as the ratio to total protein and are mean ± SEM. Each western blot analysis was repeated three independent times. Bcl-2 and cleaved caspase-3 were tested only at 120 and 140 h of HG exposure as the changes in miR-320 and the upstream target IGF-1 was not observed until 96 h. Other time points for IGF-1 are shown in Figure S2A. (E) Quantitative scatterplot bar graphs showing caspase-3/7 activity after normalizing the cell numbers using CyQUANT assay. Data are represented as arbitrary units (a.u.) and are mean ± SEM. (F) Quantitative scatterplot bar graphs showing absolute number of cells by CyQUANT assay and represented as mean ± SEM. ∗p < 0.05, ∗∗p < 0.01, ∗∗∗p < 0.001, and ∗∗∗∗p < 0.0001.

Next, to confirm that the changes observed in the expression of miR-320 originated from the cardiomyocytes and to design a therapeutic strategy for inhibition of miR-320, human ventricular cardiomyocytes (AC-16 cells) were exposed to HG to mimic the diabetic condition. Results supported findings from the whole heart, demonstrating a significant upregulation of miR-320 in cardiomyocytes and that these changes were only observed in the late stage of HG exposure (p = 0.0040 versus normal glucose [NG] at 96 h and p = 0.0001 versus NG at 120 h; Figure 3A). The western blotting analysis confirmed significant downregulation of IGF-1 (p = 0.0001 versus NG; Figures 3B, S3A–S3D) and Bcl-2 (p = 0.0001 versus NG; Figures 3C and S3E), and upregulation of pro-apoptotic cleaved caspase-3 (p = 0.0078 versus NG; Figures 3D and S3F) suggesting increased apoptotic cell death. Increased caspase-3/7 activity further confirmed increased apoptosis following exposure to HG (Figure 3E). There was no difference in the number of cells that are cultured under NG or HG condition, although, as expected, there was a time-dependent difference in number of cells both in NG and HG conditions (Figure 3F). To further confirm that the origin of increased miR-320 is from cardiomyocytes, we measured the expression of miR-320 in both endothelial cells (Figure S3G) and cardiac fibroblasts (Figure S3H), which did not show any significant changes in the expression pattern of miR-320. Of note, the expression of miR-320 was very low in both endothelial cells and fibroblasts (Figures S3G and S3H).

Based on these observations, the time point just before the onset of miR-320 upregulation was selected as the optimal time point for both *in vitro* (72 h) and *in vivo* (24 weeks) miR-320 modulation.

### Inhibition of miR-320 ameliorated apoptosis in HG-treated human ventricular cardiomyocytes

**Figure 4:**
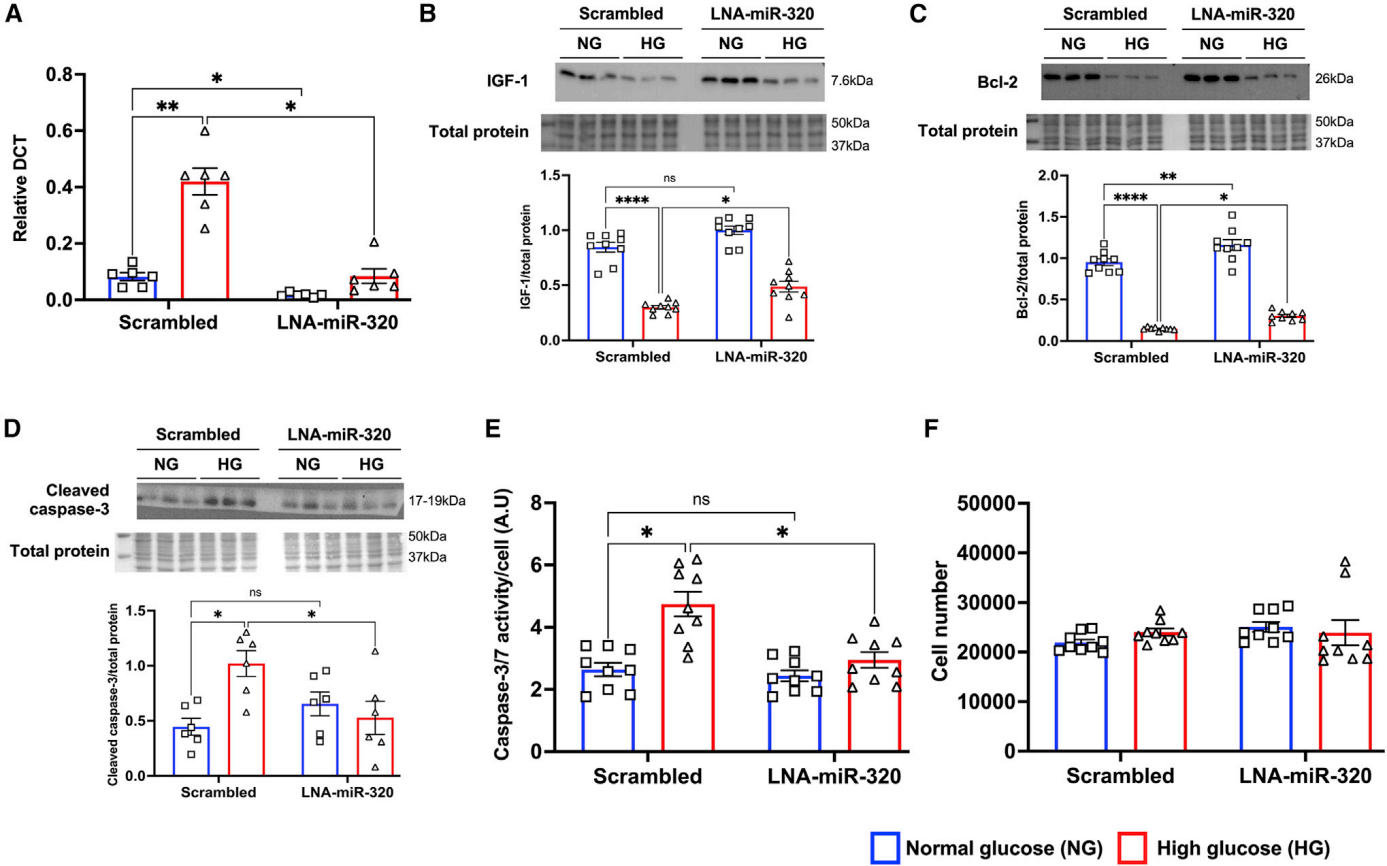
Inhibition of miR-320 ameliorated apoptosis in HG-treated human ventricular cardiomyocytes (A) Quantitative scatterplot bar graph showing the expression of miR-320 by RT-PCR analysis after transfection of AC-16 cells either LNA-precursor miR-320 (LNA-miR-320) to knock down miR-320 expression or scrambled sequence (Scrambled) as the control in both study groups. Data are mean ± SEM and expressed as relative DCT. (B–D) Representative western blots and quantitative scatterplot bar graphs showing the expression of IGF-1 (B), Bcl-2 (C), and cleaved caspase-3 (D) in both the study groups after treatment with LNA-mIR-320 or scrambled sequence. Data are represented as the ratio to total protein and are mean ± SEM. Each western blot analysis was repeated three independent times. (E) Quantitative scatterplot bar graphs showing caspase-3/7 activity after normalizing the cell numbers using CyQUANT assay. Data are represented as a.u. and are mean ± SEM. (F) Quantitative scatterplot bar graphs showing absolute number of cells by CyQUANT assay and represented as mean ± SEM. ∗p < 0.05, ∗∗p < 0.01, and ∗∗∗∗p < 0.001.

Seventy two hours after HG exposure, AC-16 cells were transfected with LNA anti-precursor miR-320 (LNA-premiR-320) or LNA-Scrambled control (LNA-Scr). RT-PCR analysis confirmed significant downregulation of miR-320 in the HG cells transfected with LNA-premiR-320 (p = 0.0001 versus LNA-Scr-treated HG cells; Figure 4A). This was reflected at the protein level with western blot analysis showing preserved IGF-1 (p = 0.0015 versus LNA-Scr-treated HG cells; Figures 4B and S4A) and Bcl-2 (p = 0.0008 versus LNA-Scr-treated HG cells; Figures 4C and S4B) expression in HG cells treated with LNA-premiR-320. Notably, this significantly reduced HG-induced apoptotic cell death, as confirmed by inhibition of cleaved caspase-3 upregulation (p = 0.0007 versus LNA-Scr-treated HG cells; Figures 4D and S4C) and reduced caspase-3/7 activity (p = 0.0006 versus LNA-Scr-treated HG cells; Figure 4E). Interestingly, knockdown of miR-320 in NG cultured cells did not show any additive increase in IGF-1 expression (Figure 4B) or caspase-3/7 activity (Figure 4B), although there was a marked upregulation of Bcl-2 (p = 0.05 versus LNA-Scr-treated NG cells; Figure 4C). Inhibition of miR-320 did not have any effect of cell numbers (Figure 4F).

### Prevention of miR-320a upregulation *in vivo* partially restored cardiac function in db/db mice

**Figure 5:**
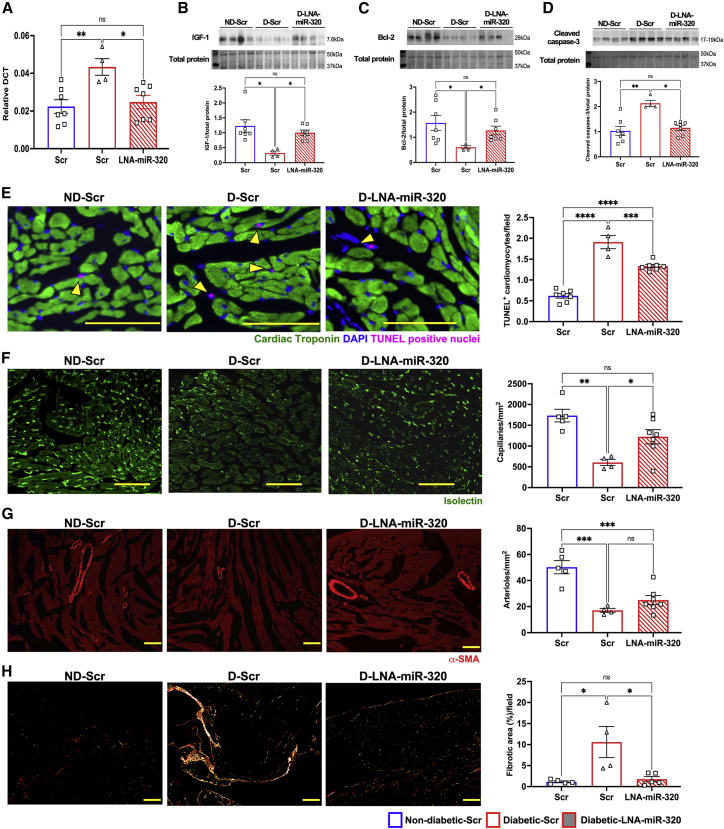
Therapeutic inhibition of miR-320 prevented adverse cardiac remodeling in type 2 diabetic db/db mice (A) Quantitative scatterplot bar graph showing the expression of miR-320 expression by RT-PCR analysis after treatment of type 2 diabetic (db/db) mice with either LNA-precursor miR-320 (LNA-miR-320) to knock down miR-320 expression or scrambled sequence (Scrambled) as the control. Lean db/+ mice were only injected with a scrambled sequence. Data are mean ± SEM and expressed as relative DCT. (B–D) Representative western blots and quantitative scatterplot bar graphs showing the expression of IGF-1 (B), Bcl-2 (C), and cleaved caspase-3 (D) in all the study groups after treatment with LNA-mIR-320 or scrambled sequence. Data are represented as the ratio to total protein and are mean ± SEM. Each western blot analysis was repeated three independent times. (E) Representative fluorescent microscopy images and the quantitative scatterplot bar graphs showing the number of TUNEL positive cardiomyocytes (arrowhead) per field. Data are mean ± SEM. (F and G) Representative confocal microscopy images and the quantitative scatterplot bar graphs show the capillaries (F, green) and arterioles (G, red) among the study groups. Data are represented as a number of capillaries (F) and arterioles (G) per mm^2^ and are mean ± SEM. (H) Representative microscopy images captured with polarized lens and the quantitative scatterplot bar graphs showing the percentage of fibrotic area per field among the study groups. Data are mean ± SEM. Five random images were taken at 200× magnification from each section, and three sections were used for each sample. Scale bars, 100 μm. ∗p < 0.05 and ∗∗p < 0.01. n = at least 4 animals in each group.

Finally, to determine if *in vitro* results are translatable *in vivo*, 24-week-old db/db mice were randomized to receive once a week intraperitoneal (i.p.) injection of LNA-scrambled (Scr) control or LNA-premiR-320 (10 mg/kg). Age-matched db/+ mice received only i.p. injection of LNA-Scr. Samples were collected at 8 weeks after the initiation of treatment. RT-PCR analysis showed no changes in the upregulated miR-320 expression in the diabetic heart treated with LNA-Scr (p = 0.0195 versus db/+ + LNA-Scr; Figure 5A). Interestingly, we observed a persistent decrease in the expression of miR-320 in db/db mice treated with LNA-premiR-320 (p = 0.0410 versus db/db + LNA-Scr; Figure 5A). The functional effect of the treatment was confirmed by preserved IGF-1 (p = 0.0423 versus db/db + LNA-Scr; Figures 5B and S5A) and Bcl-2 (p = 0.0.0410 versus db/db+ LNA-Scr; Figures 5C and S5B), and prevention of cleaved caspase-3 upregulation (p = 0.0476 versus db/db+ LNA-Scr; Figures 5D and S5C) in the LNA-premiR-320-treated db/db mice.

**Figure 6:**
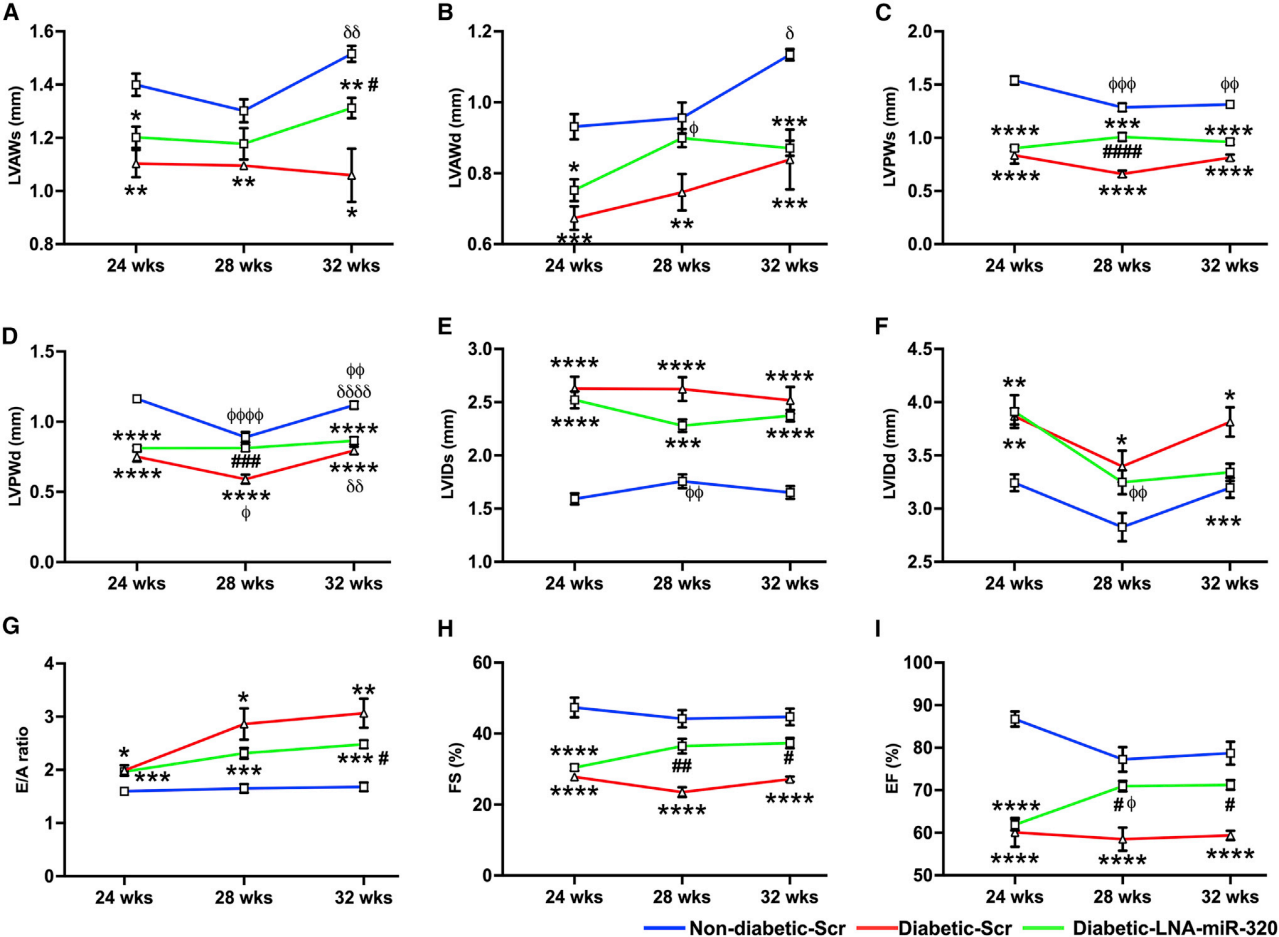
Therapeutic inhibition of miR-320 improved cardiac function (A–I) Quantitative line graphs showing cardiac functions measured by echocardiography in all the study groups. Data are mean ± SEM. LVAWs, left ventricular anterior wall during systole; LVAWd, LVAW during diastole; LVPWs, LV posterior wall during systole; LVPWs, LVPW wall during diastole; LVIDs, LV internal diameter during systole; LVIDd, LVID during diastole; FS, fractional shortening; EF, ejection fraction. ∗p < 0.05, ∗∗p < 0.01, ∗∗∗p < 0.001, and ∗∗∗∗p < 0.0001 versus non-diabetic (ND) scrambled sequence (Scr)-treated group of the corresponding age; #p < 0.05 and ##p < 0.01 versus diabetic (D) scrambled (Scr)-treated group; φp < 0.05, φφp<0.01, φφφp<0.001, and φφφφp<0.0001 versus 24 weeks (wks) age time point; δδp<0.01 and δδδδp<0.0001 versus 28 weeks time point. n = 7 animals in each group.

The histological analysis supported the molecular analysis data showing a significant increase in TUNEL-positive nuclei in the db/db heart treated with Scr (0.6193% ± 0.05339% in db/+ + LNA-Scr versus 1.909% ± 0.1583% in db/db + LNA-Scr, p = 0.0001; Figures 5E and S6), which was reduced following treatment with LNA-premiR-320 (1.333% ± 0.04138%, p = 0.0004 versus db/db + LNA-Scr; Figures 5E and S6). Vascular density analysis showed a significant improvement in the capillary density following miR-320 knockdown (1,223 ± 281/mm^2^ in db/db + LNA-premiR-320 versus 454 ± 146/mm^2^ in db/db + LNA-Scr, p = 0.04; Figures 5F and S7). While the density of small arterioles (<50 μm) showed a trend toward increase following the miR-320 knockdown, this was not significant (25 ± 9/mm^2^ in db/db + LNA-premiR-320 versus 17 ± 3/mm^2^ in db/db + LNA-Scr, p = 0.37; Figures 5G and S8). Furthermore, picrosirius red staining showed a marked reduction in structural remodeling of the diabetic heart as demonstrated by reduced interstitial fibrosis following treatment with LNA-premiR-320 (p = 0.02 versus db/bd + LNA-Scr-treated group; Figure 5H). Altogether, this improved both systolic and diastolic function in the db/db mice (Figure 6).

## Discussion

Our results have demonstrated a causal effect of miR-320 on increased apoptosis in the diabetic heart. Furthermore, we also showed that therapeutic knockdown of miR-320 in db/db mice prevented the downregulation of IGF-1, the direct target for miR-320, thereby preserving the downstream anti-apoptotic Bcl-2 signaling cascade to reduce apoptotic cell death and hence fibrotic remodeling. Overall, these resulted in improving cardiac function.

Wang et al. showed a positive correlation between miR-320 expression and HG exposure using neonatal cardiomyocytes. They also demonstrated that miR-320 released from HG-cultured cardiomyocytes induced endothelial cells dysfunction *in vitro*.^22^ While these seminal pieces of evidence demonstrated upregulation of miR-320 in response to HG,^25^, ^26^, ^27^ our finding is important because, using myocardial tissue samples collected at different time points, we were able to demonstrate that miR-320 upregulation occurs at the late stage in the diabetic heart. This is crucial for designing the therapeutic strategy for knockdown or inhibiting miR-320 activity. A recent study showed that miR-320 could induce insulin resistance, affecting glucose transporter-4 (GLUT-4) in the cardiomyocytes to suppress glucose uptake.^26^ Interestingly, our recent study showed marked downregulation of GLUT-4 in the late stage of diabetes, leading to reduced glucose uptake in the db/db mouse heart.^28^ Although we did not measure the direct effect of miR-320 on glucose uptake, based on our earlier finding we speculate that upregulated miR-320 affected GLUT-4 expression, which reduced glucose uptake in the diabetic heart.

Cardiomyocytes are highly susceptible to apoptosis in diabetic conditions.^20^ Several mechanisms have been postulated as the reason behind the increased apoptosis of diabetic cardiomyocytes. Among these, miRNAs are the key molecular mediators of cardiomyocyte apoptosis.^29^ Tian et al. demonstrated a crucial role for miR-320 in inducing cardiomyocyte apoptosis in a mouse model of myocardial ischemia-reperfusion injury.^30^ In another study, Cao and Chai demonstrated that reduced ischemia-reperfusion induced apoptosis following prevention of miR-320 upregulation by pharmacological preconditioning with morphine.^31^ IGF-1, the target gene of miR-320, is a major survival growth factor that exhibits anti-apoptotic effects in the heart.^32^ IGF-1 inhibits cardiomyocyte apoptosis by activating the downstream Bcl-2, which attenuates the induction of pro-apoptotic Bax and caspase-3. Our study showed a negative correlation between miR-320 and IGF-1 in the diabetic heart (data not shown) and, importantly, that this was associated with apoptotic cell death. Our *in vitro* studies further confirmed the causal role of miR-320 on IGF-1 downregulation and cardiomyocyte apoptosis, where upregulation of miR-320 preceded the downregulation of IGF-1, which in turn preceded cardiomyocyte apoptosis. Importantly, therapeutic knockdown of miR-320 stopped these chains of events, thereby suggesting a critical role of miR-320 in diabetic cardiomyocytes.

Type 2 diabetes is associated with a more than 2-fold increase in the development of both diastolic and systolic dysfunction.^33^, ^34^, ^35^ Increased cardiomyocyte apoptosis is one of the major contributors to the development of cardiac dysfunction.^36^ Loss of cardiomyocytes due to apoptosis results in fibrotic scar tissue formation, which, along with cardiac hypertrophy, makes the left ventricle stiff, a hallmark of diastolic dysfunction. The progression of the disease leads to systolic dysfunction.^37^ This was supported by evidence from recent studies demonstrating a direct correlation between apoptosis and ejection fraction in patients with myocardial infarction.^38^^,^^39^ In another study, reduction of apoptosis in a rat model of pressure overload markedly improved left ventricular fractional shortening.^40^

Interestingly, in our study, upregulation of miR-320 was observed only after the development of systolic dysfunction, suggesting that miR-320 does not initiate systolic dysfunction in the diabetic heart. However, increased apoptosis and fibrosis as a consequence of miR-320 upregulation is likely to have aggravated further deterioration of cardiac function, although whether miR-320 has a direct effect on fibrosis is not known. Furthermore, our study findings are contrary to a recent study that demonstrated upregulation of miR-320 expression before the development of systolic dysfunction in high fat diet-induced diabetic mice.^41^ While the reason for the discrepancy in the results between the two studies is unknown, it is likely due to the difference in the model and underlying pathology to induce diabetes.

Our previous study identified the development of vascular rarefaction from 20 weeks of age in db/db mice.^19^ In another study, we showed concurrent cardiac and vascular dysfunction development as a direct result of diabetes.^42^ In the current study, miR-320 knockdown partially restored the lost capillaries in the diabetic heart. While miR-320 is predominantly expressed in cardiomyocytes, studies have demonstrated minimal expression of miR-320 in myocardial microvascular endothelial cells, where they are predicted to target angiogenic genes, such as fetal liver kinase-1, vascular endothelial growth factor-c, and fibroblast growth factors.^22^ We also confirmed expression of miR-320 in endothelial cells, although there was no effect with HG. Previous study showed that miR-320 encapsulated in exosomes are released from cardiomyocytes and taken up by the microvascular endothelial cells to produce an anti-angiogenic effect.^43^ This could have been the case in our study. Due to the focus of this study on cardiomyocytes, we did not explore in detail the underlying mechanisms for vascular improvement. Future mechanistic studies to determine the underlying pathways will be beneficial.

In conclusion, our study identified miR-320 as the late-responding miRNA in the diabetic heart. Although miR-320 do not initiate cardiac systolic or diastolic dysfunction, increased apoptosis and fibrosis due to miR-320 upregulation aggravated further deterioration of cardiac function. Importantly, therapeutic knockdown of miR-320 both *in vitro* and *in vivo* partially reversed diabetes-induced cardiac dysfunction. While AC16 cells are valuable as a model of *in vitro* cardiomyocytes due to the proliferative nature of these cells, we acknowledge that there may be a few differences in the metabolism compared with the primary cardiomyocytes.

Our approach to therapeutical knockdown of miR-320 *in vivo* is clinically relevant as we initiated the treatment after the onset of cardiac dysfunction. In clinical practice, most patients are currently diagnosed after developing cardiac dysfunction. Therefore, the ability to partially restore the deteriorated cardiac function through inhibition of apoptosis and fibrosis and restoration of microvasculatures in the diabetic heart further emphasizes the pathological role of miR-320 in the induction of cardiac dysfunction at the later stage of the disease. Hence, therapies targeting miR-320 are highly promising for the treatment of NiDHD.

## Material and methods

### Ethics

The human myocardium study was approved by the Health and Disability Ethics Committee of New Zealand and the Human Ethics Committee at the University of Otago, Dunedin, New Zealand (approval nos. LRS/12/01/001 and HDEC#16/NTB/219). All the patients provided written consent for the collection and use of samples in this study. Collection and use of the human samples conformed to the Declaration of Helsinki. All animal experiments were approved by the Animal Ethics Committee (approval no. AUP-18-205), University of Otago, New Zealand.

### Experimental models and sample collection

#### Human myocardial tissue collection

RAA biopsies were collected from IHD patients with no history of diabetes (non-diabetic-IHD; ND-IHD, n = 12) and with a history of diabetes (diabetic-IHD; D-IHD, n = 12), who underwent on-pump coronary artery bypass graft surgery at Dunedin hospital, New Zealand (Table 1). In addition, to determine if ischemia alone had any effect of ischemia on miR-320, myocardial tissue samples collected from cadavers without any known history of ischemia or diabetes (ND-NIHD, n = 11) served as healthy controls. Myocardial samples were used from cadavers as we did not have access to the RAA. Moreover, our previous study showed no difference in the expression of miRNA between RAA and myocardium.^44^

**Table 1:** Patient characteristics

Sample ID	Age (years)	Sex	Diabetes duration (years)	Hypertension	Glucose level (Mmol/L)	HbA1c (mmol/mol)	Medication
96	51	M	N/A	No	7.2	47	No
112	72	M	N/A	No	6.5	38.8	No
114	55	M	N/A	Yes	6.0	35.3	metoprolol and atorvastatin
145	77	M	N/A	Yes	N/A	N/A	amlodipine, metoprolol, and simvastatin
156	75	M	N/A	Yes	N/A	N/A	cilazapril, metoprolol, and simvastatin
167	66	M	N/A	Yes	5.5	35	metoprolol and atorvastatin
118	68	M	N/A	Yes	N/A	N/A	metoprolol and atorvastatin
189	70	M	N/A	No	5.4	39	atorvastatin
193	52	M	N/A	Yes	5.4	34	metoprolol and atorvastatin
196	60	M	N/A	Yes	5.5	35	metoprolol
202	76	M	N/A	Yes	6.4	38.1	amlodipine, metoprolol, and atorvastatin
211	68	M	N/A	Yes	4.4	34	metoprolol and simvastatin
197	75	M	13	Yes	8.5	52.6	bisoprolol, atorvastatin, metformin, and glipizide
335	61	M	10	Yes	12	49	amlodipine, cilazapril, metformin, hydrochlorothiazide, and glipizide
340	76	M	11	Yes	10	53	cilazapril, nadolol, simvastatin, and glipizide
438	56	M	8	Yes	13.1	57	metoprolol, glipizide, and atorvastatin
439	72	M	13	Yes	6.4	61	cilazapril, metoprolol, glipizide, and atorvastatin
521	71	M	14	Yes	14	54	cilazapril, metoprolol, glipizide, and atorvastatin
543	55	M	9	Yes	9.3	56	amlodipine, bisoprolol, insulin, and atorvastatin
630	68	M	14	Yes	8.6	68	quinapril, carvedilol, glipizide, atorvastatin, and aspirin
632	72	M	16	Yes	13.8	65	cilazapril, metoprolol, metformin, aspirin, and atorvastatin
633	61	M	9	Yes	7	50	quinapril, carvedolol, metformin, atorvastatin, and aspirin
642	67	M	12	Yes	11.2	53	metformin, glipizide, aspirin, simvastatin, and bisoprolol
715	57	M	10	Yes	13	50	amlodipine, cilazapril, metoprolol, glipizide, aspirin, and atorvastatin

### Animal model of type 2 diabetes

In-house bred obese C57BL/KsJ-lepr^db^/lepr^db^ (db/db) and age-matched lean control heterozygotes (db/+) mice (both male and female sex) were used as the model of type 2 diabetes and age-matched controls, respectively.^45^ Animals were housed at the optimal room temperature of 21°C ± 1°C under a 12:12-h light-dark cycle. All the animals had access to water and a standard chow diet ad libitum. Our previous study showed the development of diastolic dysfunction in db/db mice from 20 weeks of age, which evolved into systolic dysfunction by 24 weeks.^46^ To determine the expression pattern of miR-320 and its target protein IGF-1, myocardial samples were collected under terminal anesthesia (2,2,2-tribromoethanol, 640 mg/kg i.p. injection) from both db/db and db/+ mice at 8, 12, 16, 20, 24, 28, and 32 weeks of age.

### Human ventricular cardiomyocyte (AC-16 cells) culture and HG exposure

AC-16 cells were purchased from Davidson Laboratory at the University of Colombia, New York.^47^ Cells were maintained in Dulbecco’s modified Eagle medium: Nutrient Mixture F12 (DMEM/F12) (Life Technologies, New Zealand) with 12.5% fetal bovine serum (FBS) (Thermo Fisher Scientific, New Zealand) and 1% penicillin-streptomycin solution (Thermo Fisher Scientific, New Zealand) at 37°C with a gas mixture of 5% CO_2_ with 95% air. Cells were cultured in DMEM with 5.5 mM glucose (NG, Thermo Fisher Scientific, New Zealand) for at least four passages before exposing them to high D-glucose (HG; 30 mM, Sigma-Merck, New Zealand) for 24 to 144 h to simulate *in vitro* diabetic condition. As the osmotic control, cells cultured in NG were supplemented with D-mannitol (30 mM, Sigma-Merck, New Zealand). Cells were seeded at 2.5 × 10^4^ cells per well in 12-well plates for RNA extraction, 4 × 10^3^ cells per well in 96-well plates for caspase-3/7 activity assay, and at 3 × 10^5^ cells per 60-mm dish for protein extraction. Samples for RNA and protein extraction were collected at 24, 48, 72, 96, 120, and 144 h following exposure to HG. Caspase-3/7 activity was measured at 144 h.

Experiments using human umbilical vein endothelial cells and human cardiac fibroblasts are detailed in the online supplemental information.

### Therapeutic modulation of miR-320

#### *In vitro* knockdown of miR-320

To determine if *in vitro* knockdown of miR-320 reverse HG-induced apoptosis in cardiomyocytes, AC-16 cells (3 × 10^6^ cells/well in a 6-well plate) were exposed to 72 h of HG. Following this, cells were transfected with LNA-premiR-320 or LNA-Scr (both from QIAGEN, Germany) using Lipofectamine RNAiMAX (Thermo Fisher Scientific, USA). In brief, 5 pmol LNA-premiR-320 or LNA-Scr was mixed with 5 μL Lipofectamine RNAiMax Transfection Reagent (Thermo Fisher Scientific, USA) in 200 μL DMEM without serum (Thermo Fisher Scientific, USA) and incubated for 15 min at room temperature. Then, the complex was added to the cells and swirled carefully for even distribution over the entire plate surface. We used LNA against precursor miR-320 to knockdown mature miR-320 expression rather than inhibiting the activity of mature miR-320 using anti-miR. At 120 h of HG treatment, the cell lysates were collected for RNA and protein extraction along with the measurement of caspase-3/7 activity.

#### *In vivo* knockdown of miR-320

Following baseline echocardiography (GE Vivid E9) measurement, 24-week-old db/db mice (n = 7 in each group) were randomized to receive once a week i.p. injection of LNA- Scr control or LNA-premiR-320 (10 mg/kg, both from QIAGEN, Germany). Age-matched db/+ mice received only i.p. injection of LNA-Scr, as they did not show any difference in miR-320 expression.^48^ Cardiac function was measured every 4 weeks using a Vivid E9 cardiovascular ultrasound system (GE Vingmed Ultrasound, Horten, Norway). Left ventricular wall thickness, systolic function (internal diameters, ejection fraction, fractional shortening) and diastolic dysfunction (E/A ratio, deceleration time [DecT]) were assessed as described previously by us.^18^^,^^28^^,^^49^ Animals were initially planned to receive 8 weeks of consecutive injections. However, the injection had to be terminated after 4 weeks because of a nationwide lockdown due to the COVID-19 pandemic (March 2020). In addition, previous studies have shown the effects of a single dose of LNA injection on miRNA inhibition. Therefore, we decided to continue monitoring the animals until the desired study period. At the end of 8 weeks after the first injection, following echocardiography measurement, under terminal anesthesia, the hearts were quickly removed, washed in phosphate-buffered saline, and dissected into three sections from apex to base for molecular (RNA and protein) and histological analysis.

### Expression analysis

#### Total RNA isolation and real-time qRT-PCR analysis

Total RNA from heart tissue (human and mouse) and AC-16 cells were isolated using QIAzol reagent as per the manufacturer’s instructions.^50^ Twenty nanograms of total RNA was reverse transcribed using miR-320-specific stem-loop structure reverse transcription primer (Thermo Fisher Scientific, New Zealand), followed by amplification. U6 small-nuclear RNA was used as the internal control. Relative expression was expressed as 2^−ΔCT^.

#### Western blot analysis

Cardiac tissue (human and mouse) and AC-16 cells were homogenized in ice-cold RIPA lysis buffer supplemented with a 1% protease inhibitor cocktail (Sigma-Aldrich, USA).^51^ For human tissues, due to the small size (5 mg) of samples in the ND-NIHD group, protein expression was restricted to only ND-IHD and D-IHD groups. Total protein was quantified using the Bradford protein assay (BioRad, New Zealand). Twenty or 40 μg of protein were resolved by 15% SDS-PAGE and transferred to a polyvinylidene fluoride membrane. After blocking and confirming the successful transfer of proteins using Ponceau staining, membranes were probed with primary antibodies against IGF-1 (1:1,000 dilution, BioRad), B cell lymphoma-2 (Bcl-2; 1:1,000, Cell Signaling Technology), and cleaved caspase-3 (1:500 dilution, Cell Signaling Technology) overnight at 4°C. Goat anti-rabbit secondary antibody (1:10,000 dilution, Abcam) was used for detection. The density of bands was analyzed using ImageJ (NIH, USA) software and normalized to total protein as detected using Ponceau staining.

### Functional analysis

#### Caspase-3/7 activity assay

At the end of the treatment period, cells seeded in the 96-well plate were washed with PBS and supplemented with a mixture consisting of 25 μL of caspase reagent (Promega, USA) and 25 μL of fresh medium. The reagents were gently mixed on a plate shaker at 250 rpm for 1 min and incubated at room temperature for 30 min. Following incubation, luminescence was measured using a plate reader (SpectraMax i3x; Molecular Devices, USA). Next, 50 μL of 2× concentrated CyQUANT reagent (Thermo Fisher Scientific, New Zealand) was added to each well. The plate was then incubated at room temperature for 10 min in the dark, and fluorescence was measured using the plate reader (excitation at 480 nm and emission at 520 nm, SpectraMax i3x; Molecular Devices, USA). Caspase activity (relative luminescent units) was then normalized to the cell numbers measured using the CyQUANT reagent (relative fluorescence units) and represented as caspase activity/cell number.

#### Microvascular analysis

For histological analysis, tissue sections collected from mouse heart were fixed overnight in freshly prepared 4% paraformaldehyde, followed by cryoprotection in 30% sucrose in PBS solution. The sections were then embedded in Tissue-Tek OCT compound and frozen by placing them on cold isopentane/2-methylbutane. Frozen sections were stored at −80°C until cryosectioned. Seven-micron-thick myocardial cryosections were then probed with biotin-conjugated Isolectin-B4 (1:200 dilution, Vector laboratories, USA) and anti-α-smooth muscle actin conjugated with Cy3 (1:800 dilution, Sigma-Aldrich, USA) to detect endothelial cells (to determine the capillary density) and smooth muscle cells (to determine the arteriole density), respectively. DAPI (1:1,000 dilution, Santa Cruz Biotechnologies, USA) was used to stain the nuclei. Images were captured at 200× magnification using a confocal microscope (Nikon A1). The density of capillaries was expressed as the mean number of isolectin^+^ cells/mm^2^ of cardiac tissue. The density of arterioles (<50 μm lumen size) was expressed as the mean number of αSMA^+^isolectin^+^ cells/mm^2^ of cardiac tissue.

#### *In situ* detection of apoptosis

Apoptotic cardiomyocytes were detected in 7-μm-thick myocardial cryosections using terminal deoxynucleotidyl transferase dUTP nick end labeling (TUNEL) staining following the manufacturer’s protocol (Thermo Fisher Scientific, New Zealand). Following this, sections were probed with cardiac troponin mouse monoclonal antibody (1:100 dilution, Novus Biologicals, USA) to label cardiomyocytes and counterstained with Alexa Fluro 633 goat-anti-mouse secondary antibody (1:1,000 dilution, Thermo Fisher Scientific, New Zealand). Images were captured at 200× magnification using an Olympus B×51 fluorescence microscope. Data are expressed as the percentage of TUNEL-positive cardiomyocytes in the section.

#### Picrosirius red staining to measure cardiac fibrosis

Cardiac fibrosis was assessed by picrosirius red staining as described earlier.^52^ In brief, 7-μm-thick myocardial cryosections were fixed in acetone and stained with 1% picrosirius red for 1 h, washed in acidified H_2_O, followed by dehydration in an ascending ethanol concentration. Following staining, images were captured using a Nikon bright-field microscope fitted with a polarized lens (Nikon Ti2-E). The fibrotic area was normalized to the total tissue area and expressed as a percentage of fibrotic tissue. All data were analyzed using ImageJ software (NIH).

### Statistical analysis

All statistical analyses were performed using GraphPad Prism Software. Data are expressed as mean ± SEM. Shapiro-Wilk test was used to test the normality of data distribution. A one-way ANOVA, followed by a non-parametric Kruskal-Wallis test, was used to determine the statistical difference between (1) the miRNA expression collected from different human RAA tissues and (2) the miRNA, target protein and apoptotic marker expression, and hemodynamic parameters for LNA-treated mouse heart samples. A two-way ANOVA, followed by a Tukey’s multiple comparisons test, was used to assess the statistical difference in miRNA expression, target protein expression, and caspase-3/7 activity between normal and HG-cultured AC-16 cells at baseline and after LNA treatment. A Student’s t test was used to determine the statistical difference in target protein and apoptotic markers between diabetic and non-diabetic RAA tissue. A p value <0.05 was predetermined as the level of significance for all statistical analyses.

## Data availability

All data associated with this study are presented in the paper and supplemental information.

## Supplemental information

Document S1. Figures S1–S8 and supplemental information

Document S2. Article plus supplemental information
