# Supplementary material for: Therapeutic knockdown of miR-320 improves deteriorated cardiac function in a pre-clinical model of non-ischemic diabetic heart disease
Source: Mol Ther Nucleic Acids. 2022 Jul 13;29:330–42. doi: 10.1016/j.omtn.2022.07.007 (PMC9356207; doi:10.1016/j.omtn.2022.07.007)
Supplement: Document S1. Figures S1–S8 and supplemental — information [file mmc1.pdf]

OMTN, Volume *29*

Supplemental information

Therapeutic knockdown of miR-320 improves deteriorated cardiac function in a pre-clinical model of non-ischemic diabetic heart disease

Nilanjan Ghosh, Sonya Fenton, Isabelle van Hout, Gregory T. Jones, Sean Coffey, Michael J.A. Williams, Ramanen Sugunesegran, Dominic Parry, Philip Davis, Daryl O. Schwenke, Anirudha Chatterjee, and Rajesh Katare

Supplemental Material

Supplemental Methods

Human umbilical vein endothelial cells (HUVEC) culture and high glucose exposure

Human Umbilical Vein Endothelial Cells (HUVECs) were purchased from Lonza and cultured in EGM-2 complete media (Lonza) as per manufacturer's protocol and as described in detail in our previous study[1]. For experiments, cells (0.05 x 10^6/well in 24 well plate) were exposed to high D-glucose (HG, 30mM) or D-mannitol (NG, 30mM, used as osmotic control). Samples for RNA were collected at 24, 48, 72, 96, 120 and 144 hours following exposure to HG.

Human cardiac fibroblasts culture and high glucose exposure

The human primary cardiac fibroblasts were obtained by outgrowth from right atrial appendage (RAA) biopsy samples collected from patients undergoing coronary artery bypass graft surgery for our previous study[2,3] was used in this study. The frozen cells were plated in a T75 culture flask in RPMI 1640 medium (ThermoFisher, USA) supplemented with 10% fetal bovine serum (FBS) and antibiotics. For experiments, cells (3 x 10^5/well in 6 well plate) were exposed to high D-glucose (HG, 30mM) or D-mannitol (NG, 30mM, used as osmotic control). Samples for RNA were collected at 24, 48, 72, 96, 120 and 144 hours following exposure to HG.

References

1. Rawal, S., Munasinghe, P.E., Shindikar, A., Paulin, J., Cameron, V., Manning, P., Williams, M.J., Jones, G.T., Bunton, R., Galvin, I., and Katare, R. (2017). Down-regulation of proangiogenic microRNA-126 and microRNA-132 are early modulators of diabetic cardiac microangiopathy. Cardiovasc Res *113*, 90-101. 10.1093/cvr/cvw235.

2. Rawal, S., Munasinghe, P.E., Nagesh, P.T., Lew, J.K.S., Jones, G.T., Williams, M.J.A., Davis, P., Bunton, D., Galvin, I.F., Manning, P., Lamberts, R.R., et al. (2017). Down-regulation of miR-15a/b accelerates fibrotic remodelling in the Type 2 diabetic human and mouse heart. Clin Sci (Lond) *131*, 847-863. 10.1042/CS20160916.

3. S., Y.-G.A., A, Y., I, v.H., J, B.P., R, S., D, P., P, D., and Katare, R. (2022). Cardiac Progenitor Cells and Adipocyte Stem Cells from Same Patients Exhibit In Vitro Functional Differences. International Journal of molecular science *17*. 10.3390/ijms23105588.

Supplemental Figure 1

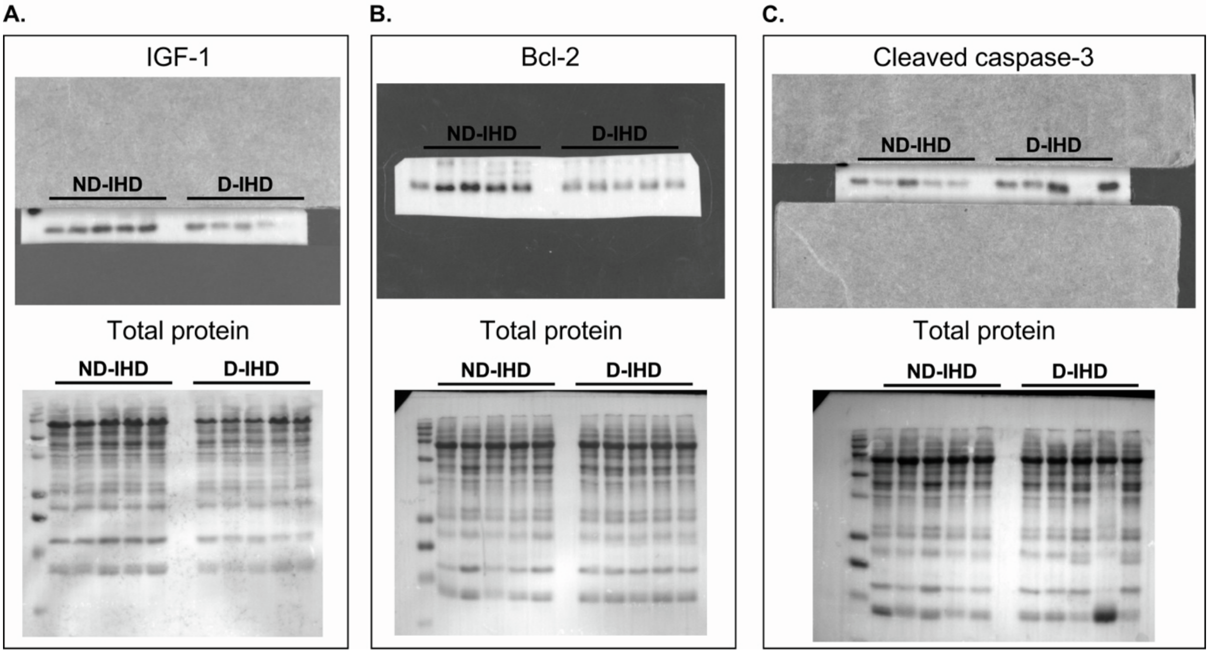

Supplemental Figure 1. Uncropped blots showing the expression of IGF-1 (**A, shown in Figure 1B**), Bcl-2 (**B, shown in Figure 1C**), and cleaved caspase-3 (CC3, **C, shown in Figure 1D**) in human RAA tissue.

Supplemental Figure 2

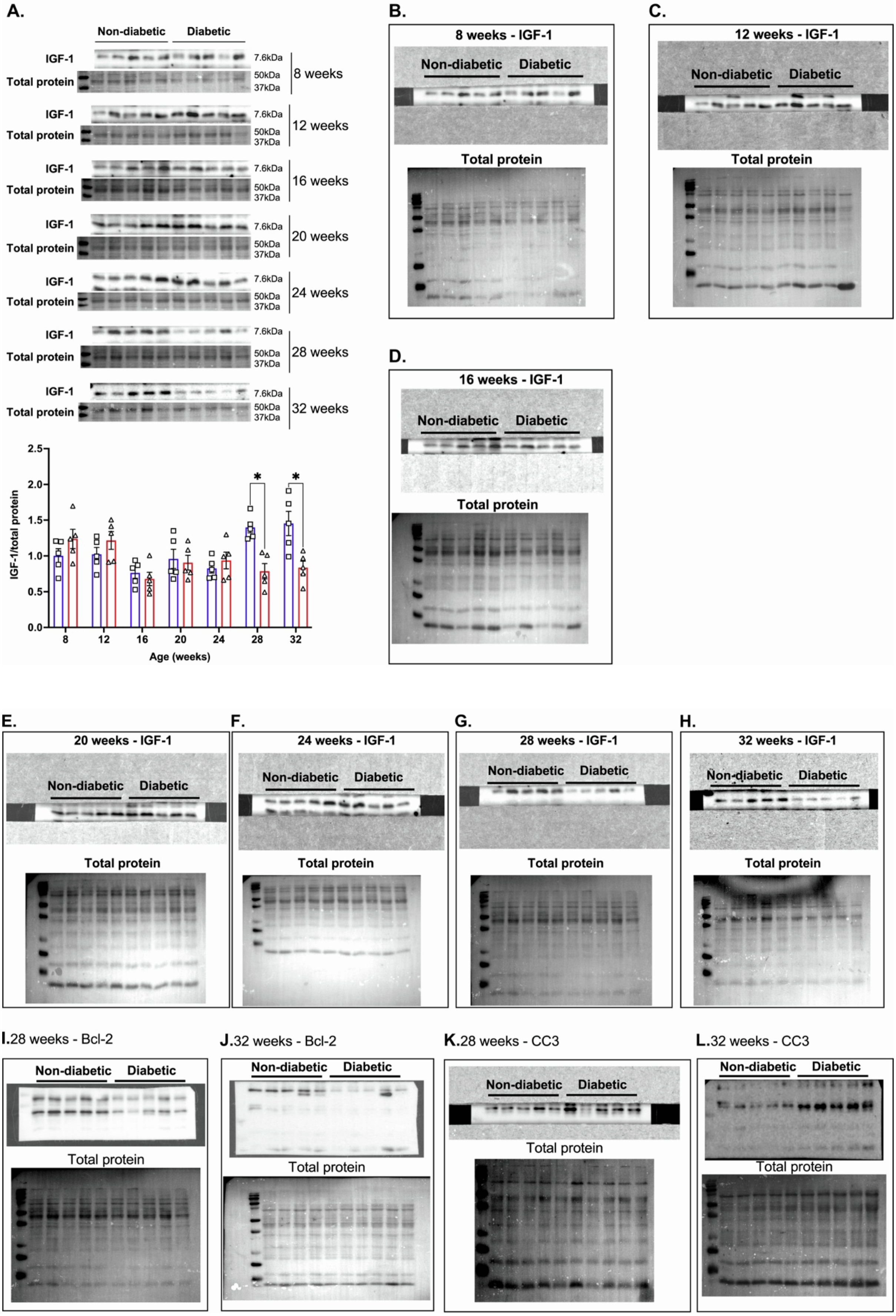

Supplemental Figure 2. A. Representative western blots and quantitative scatter plot bar graphs showing the expression of IGF-1 at different time points in the study groups. Data are represented as the ratio to total protein and are mean±SEM. Each western blot analysis was repeated three independent times. *P<0.05. **B-L.** Uncropped blots showing the expression of IGF-1 (**B-H, shown in Figure 2B and Supplemental Figure 2A**), Bcl-2 (**I&J, shown in Figure 2C**), and cleaved caspase-3 (CC3, **K&L, shown in Figure 2D**) in heart tissue collected from diabetic (db/db) and lean non-diabetic (db/+) mice.

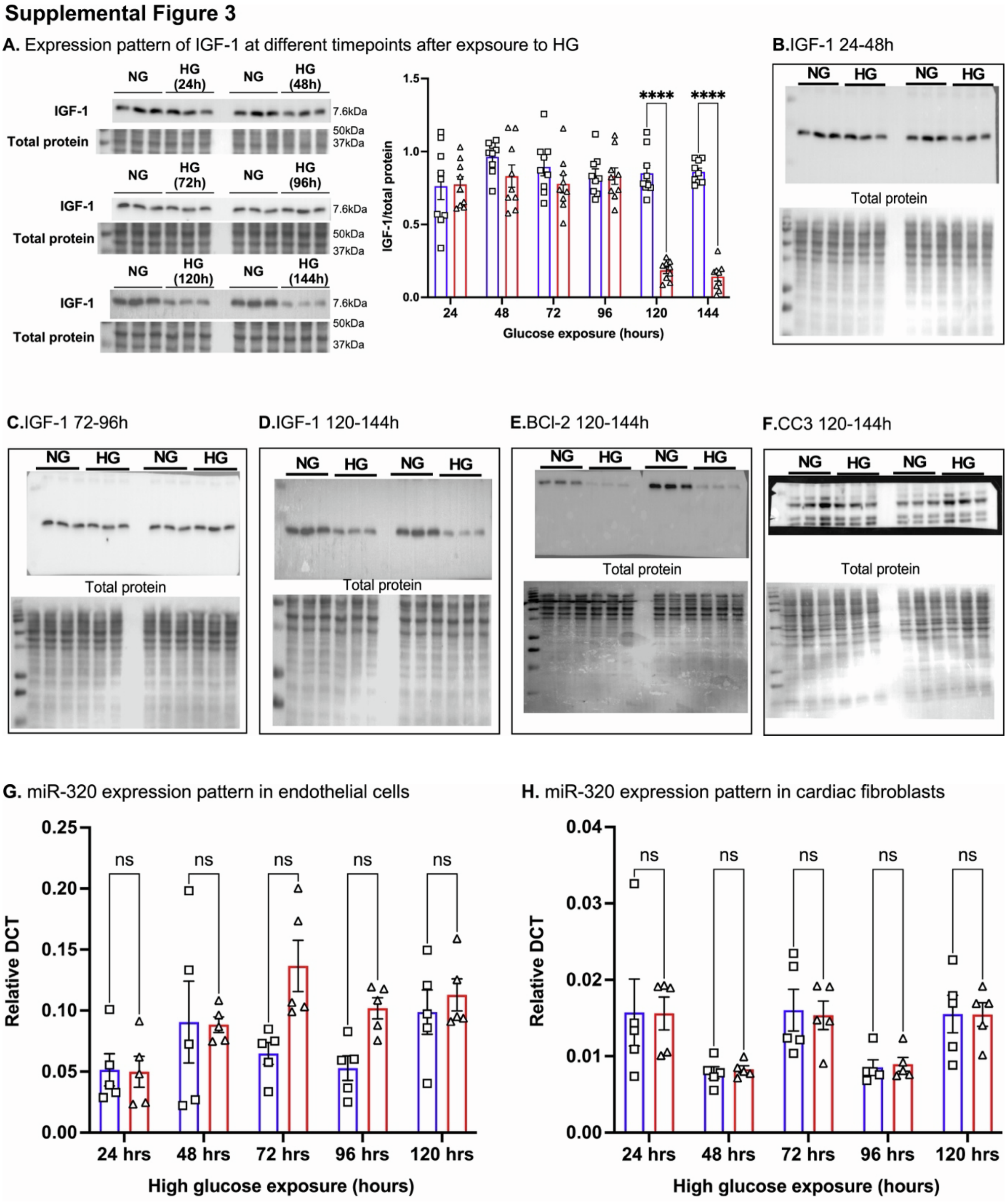

Supplemental Figure 3. A. Representative western blots and quantitative scatter plot bar graphs showing the expression of IGF-1 at different time points in the study groups. Data are represented as the ratio to total protein and are mean±SEM. Each western blot analysis was repeated three independent times. Uncropped blots showing the expression of IGF-1 (**B-D, shown in Figure 3B**), Bcl-2 (**E, shown in Figure 3C**), and cleaved caspase-3 (CC3, **F, shown**

in Figure 3D) in AC-16 human cardiomyocytes exposed to high glucose or mannitol (as osmotic control) for different time points. **G-H.** Quantitative scatter plot bar graphs showing miR-320 expression in the normal glucose (NG) and high glucose (HG) treated HUVEC (**G**) and cardiac fibroblasts (**H**) by RT-PCR analysis. Samples were collected at different points after exposing the cells to high glucose (HG) or mannitol for osmotic control (NG). Data are mean±SEM and expressed as relative DCT. n.s – not significant.

Supplemental Figure 4

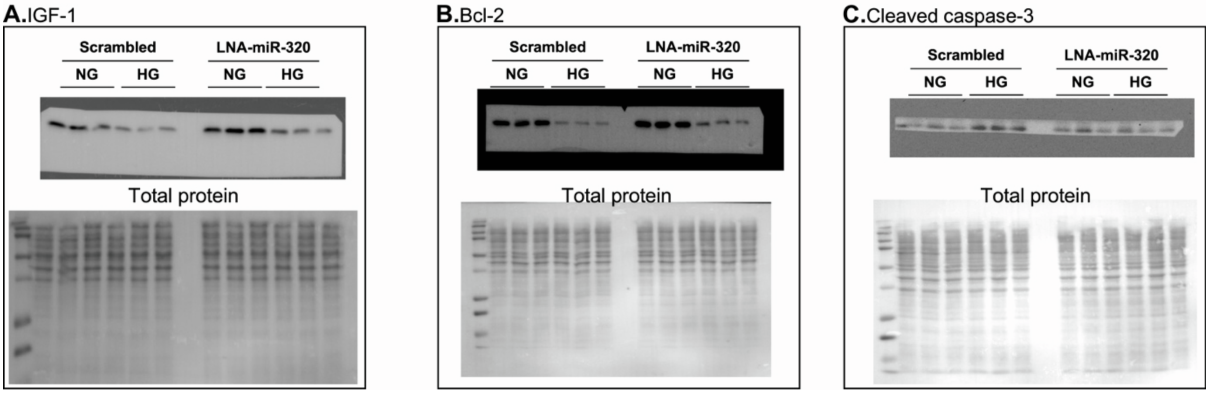

Supplemental Figure 4. Uncropped blots showing the expression of IGF-1 (**A, shown in Figure 4B**), Bcl-2 (**B, shown in Figure 4C**), and cleaved caspase-3 (CC3, **D, shown in Figure 4D**) in AC-16 human cardiomyocytes exposed to high glucose or mannitol (as osmotic control) and treated with either control scrambled sequence or LNA-miR-320 precursor to knockdown miR-320.

Supplemental Figure 5

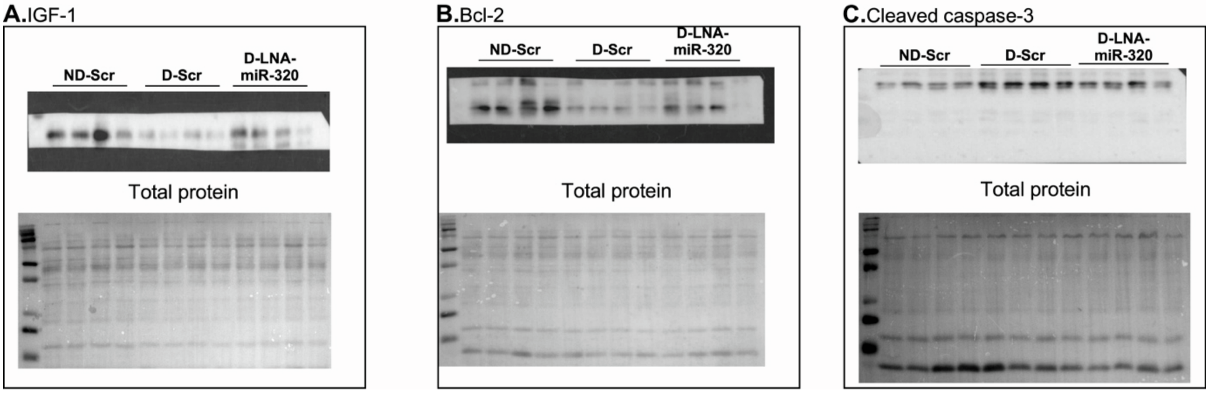

Supplemental Figure 5. Uncropped blots showing the expression of IGF-1 (**A, shown in Figure 5B**), Bcl-2 (**B, shown in Figure 5C**), and cleaved caspase-3 (CC3, **D, shown in Figure 5D**) in heart tissue collected from diabetic (db/db) and lean non-diabetic (db/+) mice following treatment with control scrambled sequence or LNA-miR-320 precursor to knockdown miR-320.

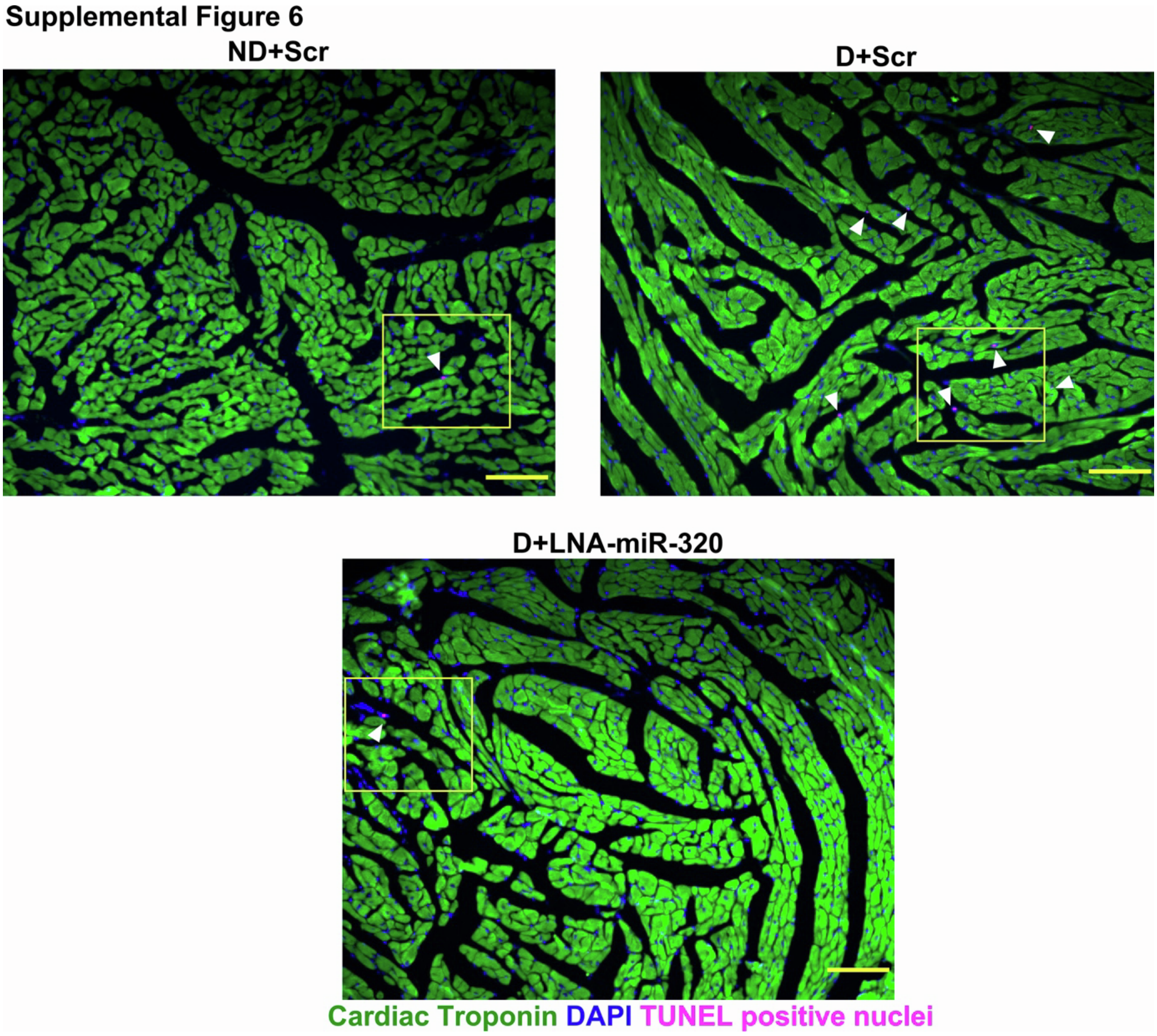

Supplemental Figure 6. Full fluorescent microscopy image of the section showing TUNEL positive (pink) cardiomyocytes (green) among the study groups. The white arrow points to the TUNEL positive cells. The yellow boxed area is zoomed in **Figure 5E**. Scale bars are 100 µm.

Supplemental Figure 7

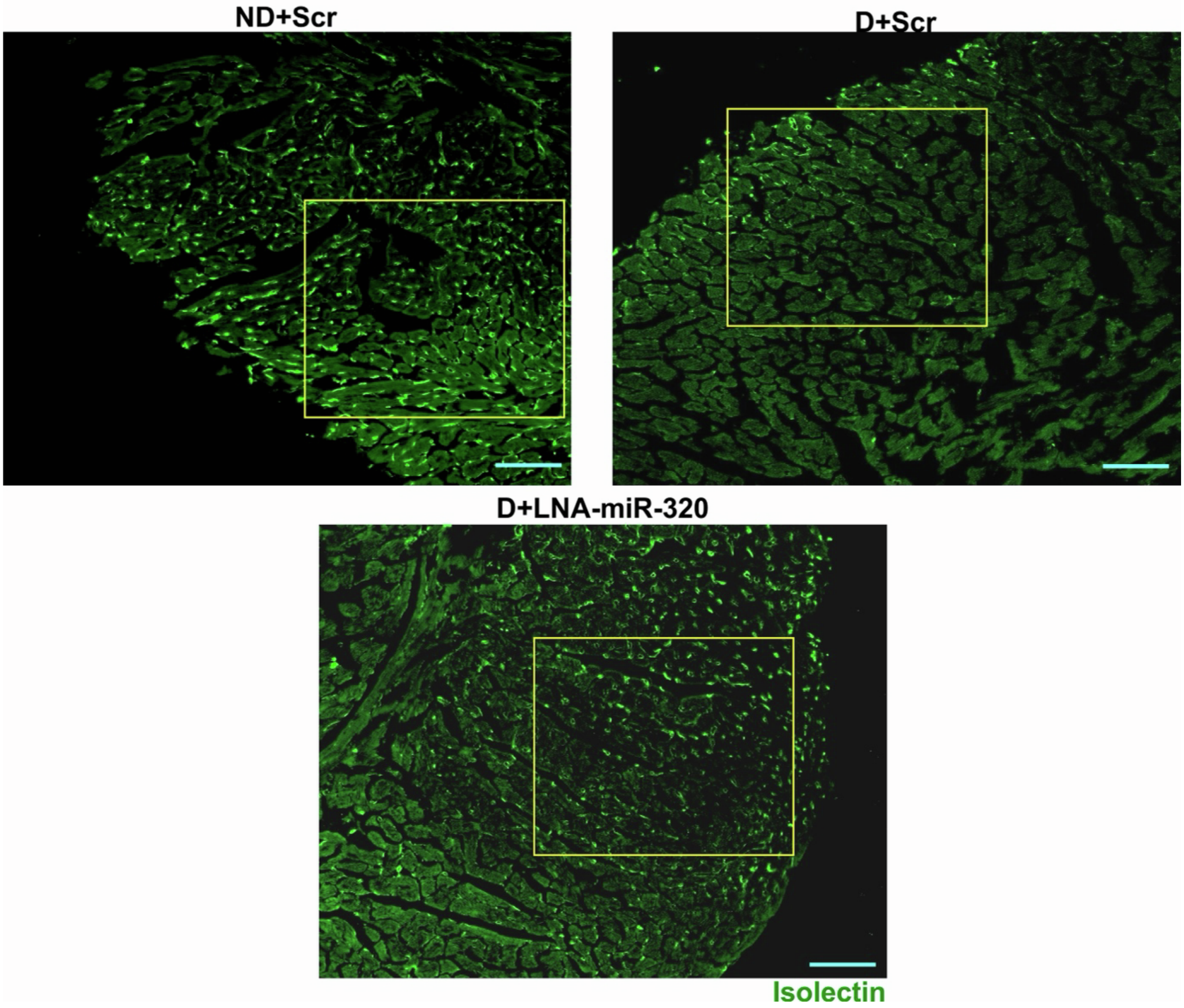

Supplemental Figure 7. Whole confocal microscopy of the section showing isolectin positive endothelial cells (green) among the study groups. The yellow boxed area is represented in **Figure 5F**. Scale bars are 100 μm.

Supplemental Figure 8

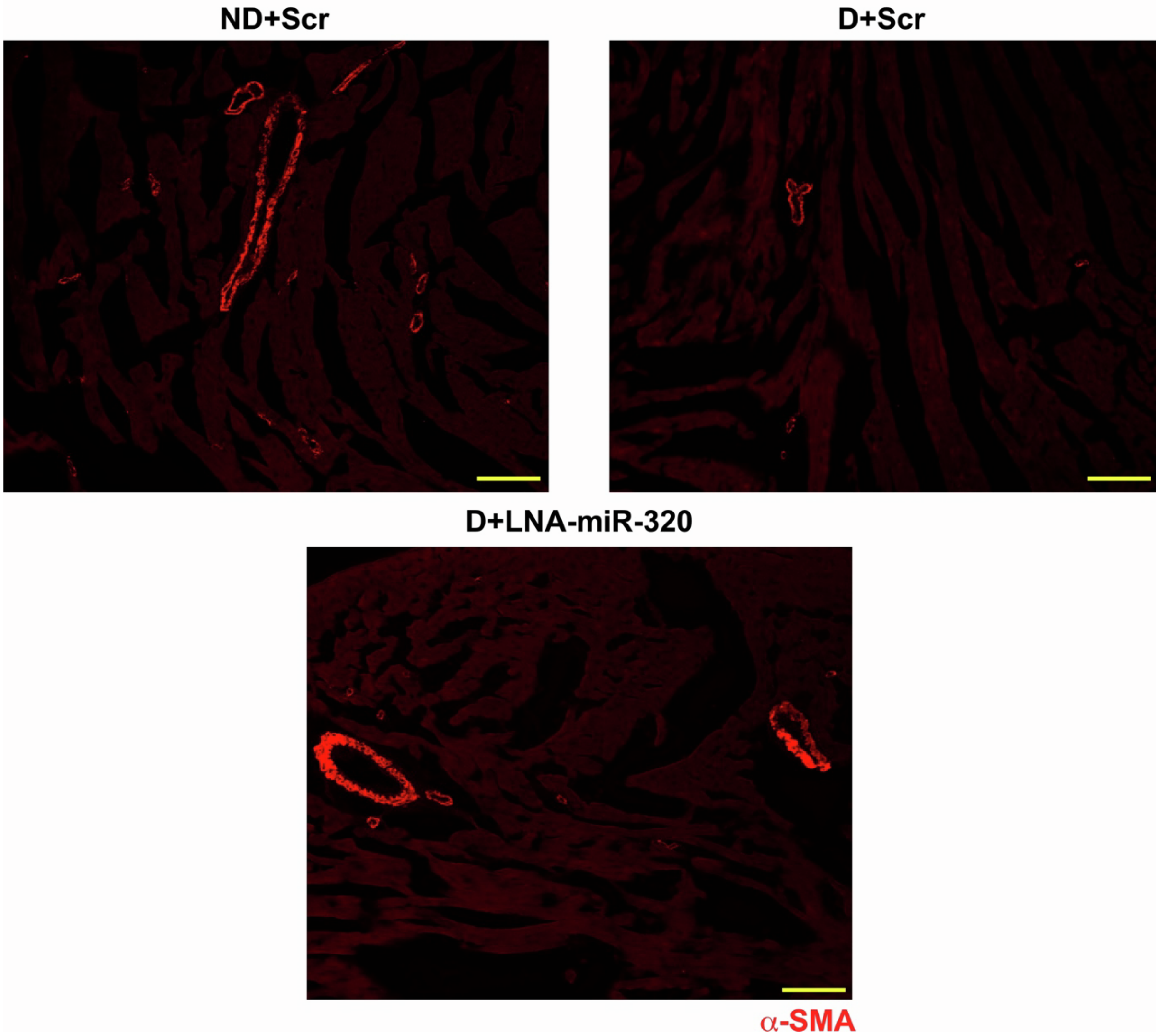

Supplemental Figure 8. Whole confocal microscopy of the section showing alpha-smooth muscle actin (α-SMA) positive cells (red) among the study groups. A smaller version of the image is represented in **Figure 5F**. Scale bars are 100 μm.
