# Supplementary material for: Therapeutic knockdown of miR-320 improves deteriorated cardiac function in a pre-clinical model of non-ischemic diabetic heart disease
Source: Mol Ther Nucleic Acids. 2022 Jul 13;29:330–42. doi: 10.1016/j.omtn.2022.07.007 (PMC9356207; doi:10.1016/j.omtn.2022.07.007)
Supplement: Document S2. Article plus supplemental information [file mmc2.pdf]

Molecular Therapy
Nucleic Acids
Original Article

Therapeutic knockdown of miR-320 improves deteriorated cardiac function in a pre-clinical model of non-ischemic diabetic heart disease

Nilanjan Ghosh,[1] Sonya Fenton,[1] Isabelle van Hout,[1] Gregory T. Jones,[2] Sean Coffey,[3] Michael J.A. Williams,[3] Ramanen Sugunesegran,[4] Dominic Parry,[4] Philip Davis,[4] Daryl O. Schwenke,[1] Anirudha Chatterjee,[5,6] and Rajesh Katare[1]

[1]Department of Physiology-HeartOtago, School of Biomedical Sciences, University of Otago, Dunedin, New Zealand; [2]Department of Surgical Sciences, University of Otago, Dunedin, New Zealand; [3]Department of Medicine, University of Otago, Dunedin, New Zealand; [4]Department of Cardiothoracic Surgery, University of Otago, Dunedin, New Zealand; [5]Department of Pathology, Dunedin School of Medicine, University of Otago, Dunedin, New Zealand; [6]Honorary Professor, UPES University, Dehradun, India

Non-ischemic diabetic heart disease (NiDHD) is characterized by diastolic dysfunction and decreased or preserved systolic function, eventually resulting in heart failure. Accelerated apoptotic cell death because of alteration of molecular signaling pathways due to dysregulation in microRNAs (miRNAs) plays a significant role in the development of NiDHD. Here, we aimed to determine the pathological role of cardiomyocyte-enriched pro-apoptotic miR-320 in the development of NiDHD. We identified a marked upregulation of miR-320 that was associated with downregulation of its target protein insulin growth factor-1 (IGF-1) in human right atrial appendage tissue in the late stages of cardiomyopathy in type 2 diabetic db/db mice and high-glucose-cultured human ventricular cardiomyocytes (AC-16 cells). *In vitro* knockdown of miR-320 in high-glucose-exposed AC-16 cells using locked nucleic acid (LNA) anti-miR-320 markedly reduced high-glucose-induced apoptosis by restoring IGF-1 and Bcl-2. Finally, *in vivo* knockdown of miR-320 in 24-week-old type 2 diabetic db/db mice reduced cardiomyocyte apoptosis and interstitial fibrosis while restoring vascular density. This resulted in partial recovery of the impaired diastolic and systolic function. Our study provides evidence that miR-320 is a late-responding miRNA that aggravates apoptosis and cardiac dysfunction in the diabetic heart, and that therapeutic knockdown of miR-320 is beneficial in partially restoring the deteriorated cardiac function.

INTRODUCTION

More than 70% of individuals with diabetes develop some form of heart disease during their life span.[1] Notably, the risk of heart failure increases 2- to 3-fold in the presence of diabetes.[2,3] The impact of diabetes on vascular and ischemic disease, such as coronary and cerebrovascular disease, is well established, but non-ischemic cardiac complications associated with diabetes have received relatively little attention. Non-ischemic diabetic heart disease (NiDHD) is a chronic complication characterized by ventricular dilation and hypertrophy, diastolic dysfunction, decreased or preserved systolic function, eventually resulting in heart failure.[4,5] While numerous factors have been associated with the development of NiDHD,[6–9] the fundamental mechanisms leading to NiDHD are still not known. Abnormal levels of glycated hemoglobin have been linked to the development of NiDHD. However, studies have demonstrated that intensive glycemic control was insufficient in reducing the risk of heart failure among patients with diabetes mellitus.[10] Accumulating evidence suggests that alteration in the molecular signaling pathways due to dysregulation in microRNAs (miRNAs) plays a crucial role in developing NiDHD.[11,12]

miRNAs are small non-coding RNAs that have well-established functions in cardiovascular diseases, cancer, and metabolic disorders and are described as the "micromanagers" of gene expression.[13] We, along with others, have identified the crucial role of miRNAs in the diabetic heart.[14–19] We demonstrated that dysregulation in miRNAs is associated with vascular rarefaction, increased fibrosis, and accelerated aging in the diabetic heart.

Cardiomyocytes are highly susceptible to apoptosis in diabetic conditions.[20] Previous studies showed that miR-320 is expressed abundantly in cardiomyocytes[21,22] and directly targets the pro-survival and anti-apoptotic gene insulin growth factor-1 (IGF-1).[22,23] IGF-1 is an important regulator that maintains myocardial structural integrity and homeostasis.[21] Overexpression of miR-320 in cardiomyocytes downregulated the expression of anti-apoptotic protein Bcl-2 while increasing the expression of pro-apoptotic proteins Bax and caspase-3, the

Received 22 March 2022; accepted 8 July 2022;
https://doi.org/10.1016/j.omtn.2022.07.007.

Correspondence: Rajesh Katare, MD, PhD, Department of Physiology, HeartOtago, University of Otago, 270, Great King Street, Dunedin 9016, New Zealand.
E-mail: rajesh.katare@otago.ac.nz

 © 2022 The Author(s).
This is an open access article under the CC BY license (http://creativecommons.org/licenses/by/4.0/).

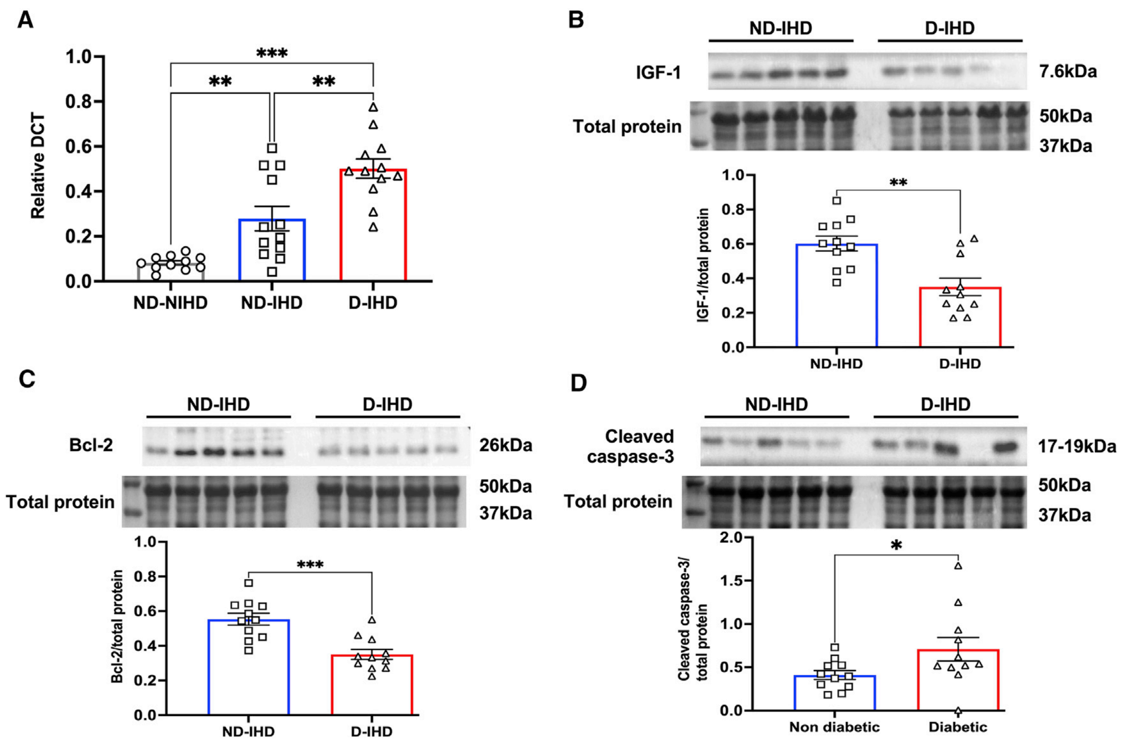

Figure 1. Diabetes upregulates miR-320 in the human heart

(A) Quantitative scatterplot bar graph showing miR-320 expression in the RAA tissue by RT-PCR analysis. Samples were collected from diabetic (D) and non-diabetic (ND) patients with ischemic heart disease (IHD) undergoing coronary artery bypass graft surgery. Data are mean ± SEM and expressed as relative DCT. (B–D) Representative western blots and quantitative scatterplot bar graphs showing the expression of IGF-1 (B), Bcl-2 (C), and cleaved caspase-3 (D) in the study groups. Data are represented as the ratio to total protein and are mean ± SEM. Each western blot analysis was repeated three independent times. *p < 0.05, **p < 0.01, and ***p < 0.001.

downstream signaling cascade of IGF-1, leading to increased cell death, suggesting a pathological role for miR-320 in the heart.[24]

In this study, we demonstrate the upregulation of pro-apoptotic miR-320 in the heart of individuals with type 2 diabetes, type 2 diabetic (db/db) mice, and in high glucose (HG)-cultured human ventricular cardiomyocytes (AC-16 cells). This was associated with the downregulation of pro-survival IGF-1, the direct target of miR-320, resulting in inhibition of its downstream anti-apoptotic signaling cascade. Importantly, *in vitro* and *in vivo* knockdown of miR-320 using locked nucleic acid (LNA) could reverse apoptotic cell death in HG-cultured AC-16 cells and db/db mice, rescuing the impaired cardiac function.

RESULTS

The uncropped western blots and full histological images are presented as figures in the supplemental information.

Diabetes upregulates miR-320 in the human heart

RT-PCR analysis showed significant upregulation of miR-320 in right atrial appendage (RAA) biopsies collected from non-diabetic (ND) patients with ischemic heart disease (IHD) compared with the hearts collected from ND cadavers without any history of diabetes or IHD (ND-NIHD) ($p = 0.006$; Figure 1A). However, diabetes-induced further upregulation of miR-320 ($p = 0.001$ versus ND-IHD; Figure 1A). Importantly, this was associated with marked downregulation of IGF-1 ($p = 0.0024$, versus ND-IHD; Figures 1B and S1A). IGF-1 is a pro-survival protein that regulates anti-apoptotic activity by regulating Bcl-2 and caspase activity. In line with this, western blot analysis showed significant downregulation of Bcl-2 ($p = 0.0006$ versus ND-IHD; Figures 1C and S1B), while apoptotic marker cleaved caspase-3 was upregulated ($p = 0.040$ versus ND-IHD; Figures 1D and S1D).

miR-320 upregulates in the later stage of diabetes

While the RT-PCR analysis confirmed that diabetes induces upregulation of miR-320 in the human heart (Figure 1A), it was not clear whether the upregulation of miR-320 is a causative factor for increased apoptosis in the diabetic heart and whether diabetes alone in the absence of ischemia is able to induce the upregulation of miR-320 that is sufficient to induce apoptosis. Therefore, to establish the mechanistic role for miR-320 in NiDHD, myocardial tissue samples were collected from 8- to 32-week-old db/db mice and their age-matched ND db/+ mice to determine the expression of miR-320 and IGF-1. RT-PCR analysis demonstrated a significant increase in miR-320 at both 28 ($p = 0.042$) and 32 weeks ($p = 0.033$) of age in db/db mice (Figure 2A). This was associated with downregulation

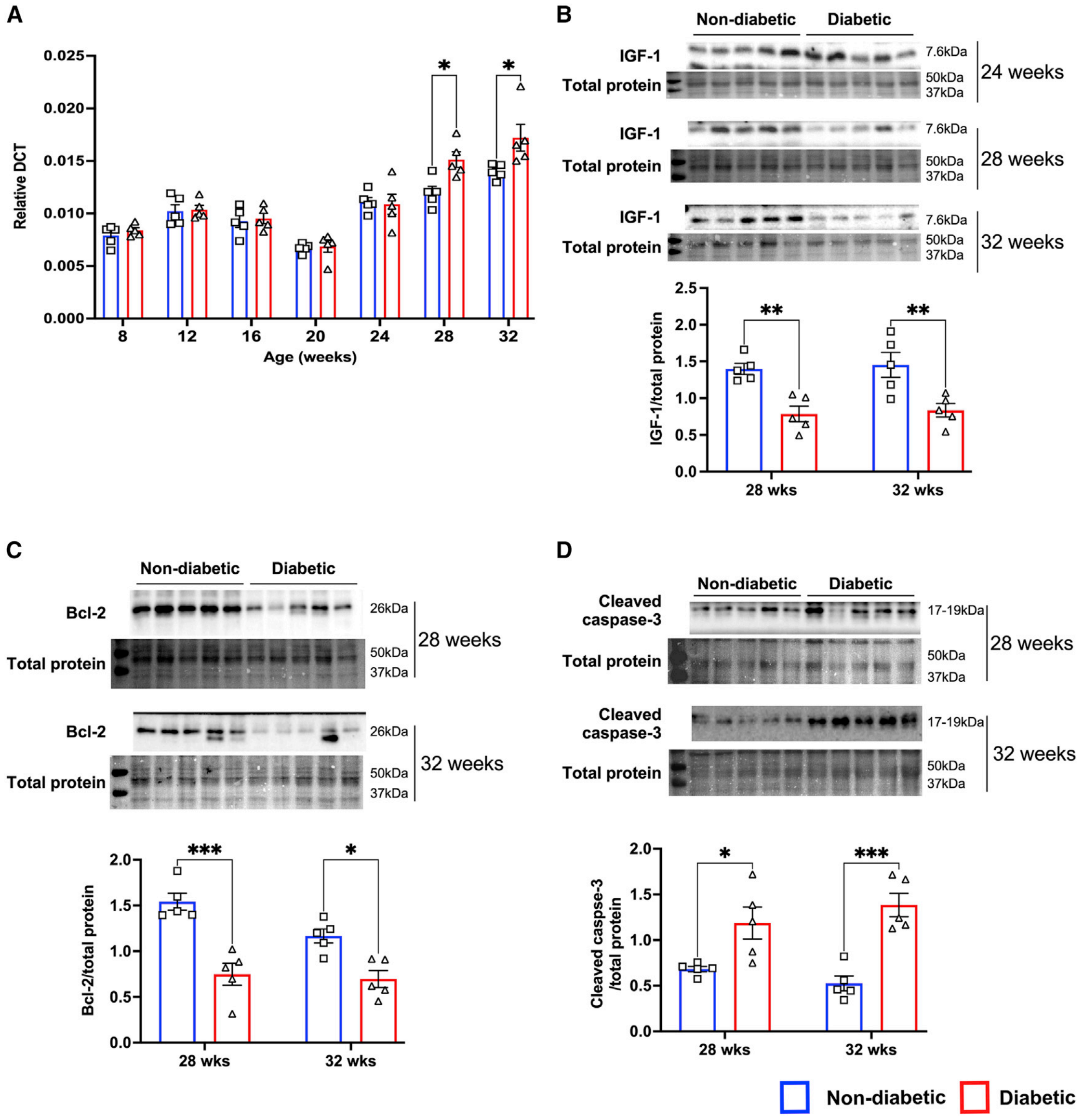

Figure 2. miR-320 upregulates in the later stage of diabetes

(A) Quantitative scatterplot bar graph showing miR-320 expression in the heart by RT-PCR analysis. Samples were collected from obese diabetic db/db mice and lean non-diabetic db/+ mice at different time points. Data are mean ± SEM and expressed as relative DCT. (B–D) Representative western blots and quantitative scatterplot bar graphs showing the expression of IGF-1 (B), Bcl-2 (C), and cleaved caspase-3 (D) at different time points in the study groups. Data are represented as the ratio to total protein and are mean ± SEM. Each western blot analysis was repeated three independent times. Bcl-2 and cleaved caspase-3 were tested only at 28 and 32 weeks as the changes in miR-320 and the upstream target IGF-1 was not observed until 28 weeks of age. Other time points for IGF-1 are shown in Figure S2A. $^{*}p < 0.05$ and $^{***}p < 0.001$. The same image was used in (B) and (C) for total proteins as the membrane used to probe IGF-1 (B) and Bcl-2 (C).

of target protein IGF-1 at both 28 ($p = 0.0138$) and 32 ($p = 0.0124$) weeks (Figures 2B and S2A–S2H). No significant changes were observed in miR-320 and IGF-1 expression from 8 to 24 weeks (Figures 2A and 2B). Western blot analysis further confirmed significant downregulation of anti-apoptotic Bcl-2 ($p = 0.0001$ at 28 weeks and $p = 0.0156$ at 32 weeks, Figures 2C, S2I, and S2J) and upregulation of cleaved caspase-3 ($p = 0.0340$ at 28 weeks and $p = 0.0005$ at 32 weeks; Figures 2D, S2K, and S2L) in db/db mice.

HG induces upregulation of miR-320 in cultured human cardiomyocytes

Next, to confirm that the changes observed in the expression of miR-320 originated from the cardiomyocytes and to design a therapeutic strategy for inhibition of miR-320, human ventricular cardiomyocytes (AC-16 cells) were exposed to HG to mimic the diabetic condition. Results supported findings from the whole heart, demonstrating a significant upregulation of miR-320 in cardiomyocytes and that

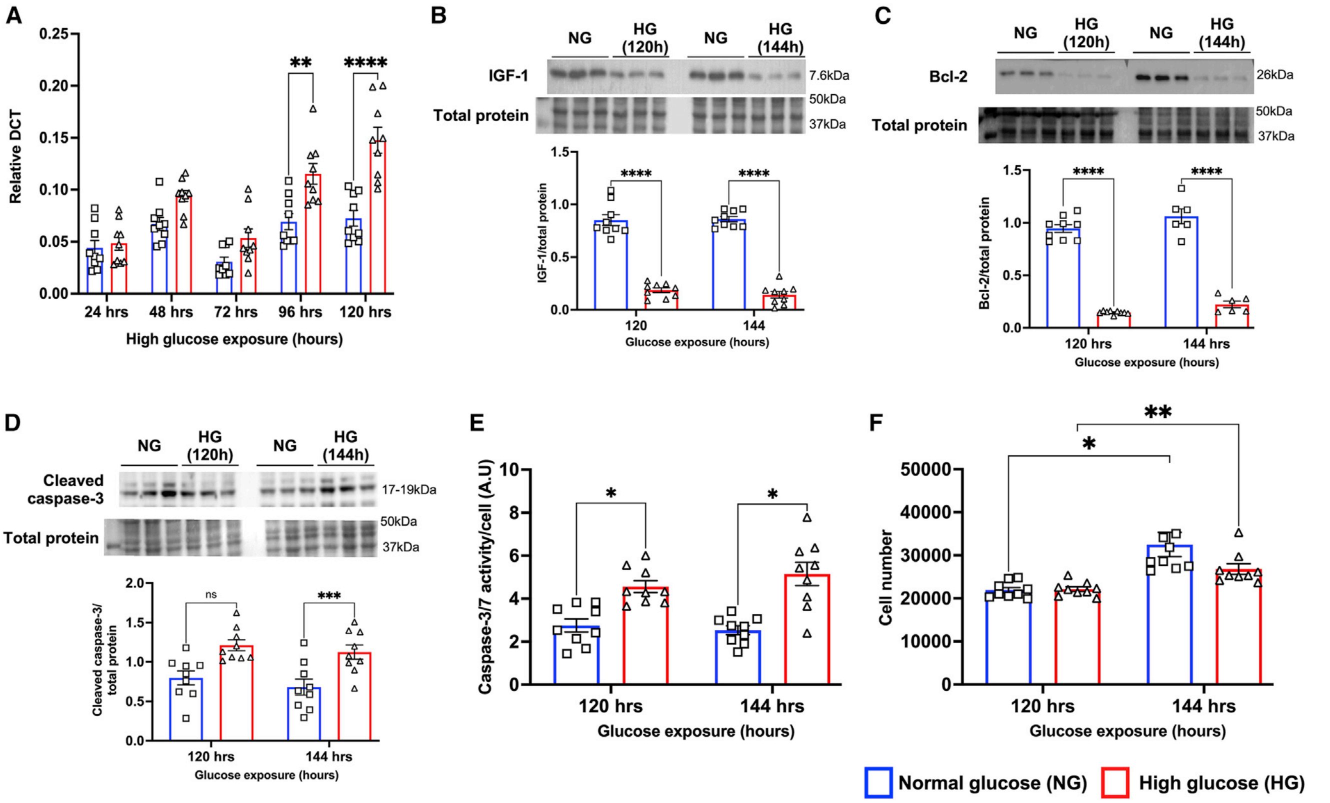

Figure 3. High glucose induces upregulation of miR-320 in cultured human cardiomyocytes

(A) Quantitative scatterplot bar graph showing miR-320 expression in the normal glucose (NG) and high glucose (HG)-treated AC-16 human cardiomyocytes by RT-PCR analysis. Samples were collected at different points after exposing the cells to HG or mannitol for osmotic control (NG). Data are mean ± SEM and expressed as relative DCT. (B–D) Representative western blots and quantitative scatterplot bar graphs showing the expression of IGF-1 (B), Bcl-2 (C), and cleaved caspase-3 (D) at different time points in the study groups. Data are represented as the ratio to total protein and are mean ± SEM. Each western blot analysis was repeated three independent times. Bcl-2 and cleaved caspase-3 were tested only at 120 and 140 h of HG exposure as the changes in miR-320 and the upstream target IGF-1 was not observed until 96 h. Other time points for IGF-1 are shown in Figure S2A. (E) Quantitative scatterplot bar graphs showing caspase-3/7 activity after normalizing the cell numbers using CyQUANT assay. Data are represented as arbitrary units (a.u.) and are mean ± SEM. (F) Quantitative scatterplot bar graphs showing absolute number of cells by CyQUANT assay and represented as mean ± SEM. *$p < 0.05$, **$p < 0.01$, ***$p < 0.001$, and ****$p < 0.0001$.

these changes were only observed in the late stage of HG exposure ($p = 0.0040$ versus normal glucose [NG] at 96 h and $p = 0.0001$ versus NG at 120 h; Figure 3A). The western blotting analysis confirmed significant downregulation of IGF-1 ($p = 0.0001$ versus NG; Figures 3B, S3A–S3D) and Bcl-2 ($p = 0.0001$ versus NG; Figures 3C and S3E), and upregulation of pro-apoptotic cleaved caspase-3 ($p = 0.0078$ versus NG; Figures 3D and S3F) suggesting increased apoptotic cell death. Increased caspase-3/7 activity further confirmed increased apoptosis following exposure to HG (Figure 3E). There was no difference in the number of cells that are cultured under NG or HG condition, although, as expected, there was a time-dependent difference in number of cells both in NG and HG conditions (Figure 3F). To further confirm that the origin of increased miR-320 is from cardiomyocytes, we measured the expression of miR-320 in both endothelial cells (Figure S3G) and cardiac fibroblasts (Figure S3H), which did not show any significant changes in the expression pattern of miR-320. Of note, the expression of miR-320 was very low in both endothelial cells and fibroblasts (Figures S3G and S3H).

Based on these observations, the time point just before the onset of miR-320 upregulation was selected as the optimal time point for both *in vitro* (72 h) and *in vivo* (24 weeks) miR-320 modulation.

Inhibition of miR-320 ameliorated apoptosis in HG-treated human ventricular cardiomyocytes

Seventy two hours after HG exposure, AC-16 cells were transfected with LNA anti-precursor miR-320 (LNA-premiR-320) or LNA-Scrambled control (LNA-Scr). RT-PCR analysis confirmed significant downregulation of miR-320 in the HG cells transfected with LNA-premiR-320 ($p = 0.0001$ versus LNA-Scr-treated HG cells; Figure 4A). This was reflected at the protein level with western blot analysis showing preserved IGF-1 ($p = 0.0015$ versus LNA-Scr-treated HG cells; Figures 4B and S4A) and Bcl-2 ($p = 0.0008$ versus LNA-Scr-treated HG cells; Figures 4C and S4B) expression in HG cells treated with LNA-premiR-320. Notably, this significantly reduced HG-induced apoptotic cell death, as confirmed by inhibition of cleaved caspase-3 upregulation ($p = 0.0007$ versus LNA-Scr-treated HG cells;

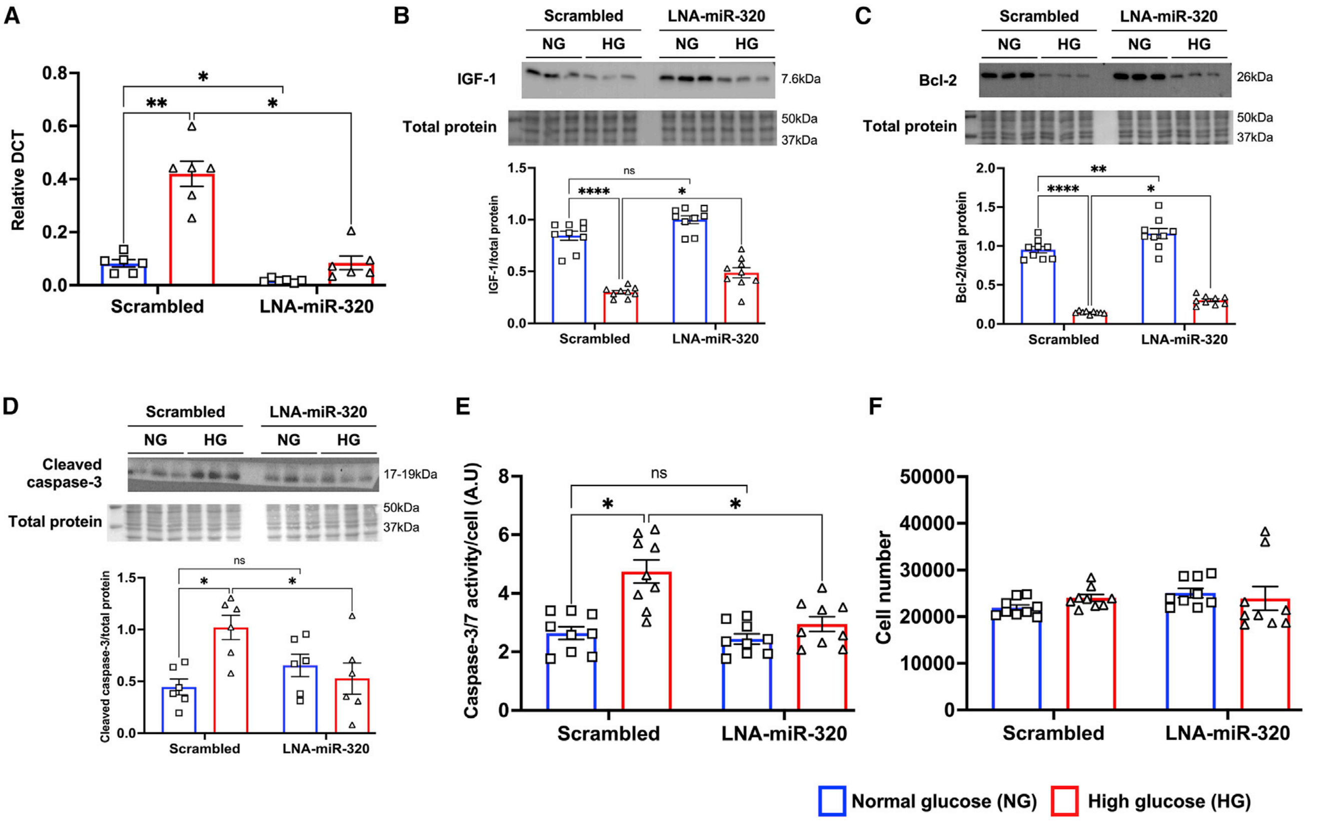

Figure 4. Inhibition of miR-320 ameliorated apoptosis in HG-treated human ventricular cardiomyocytes

(A) Quantitative scatterplot bar graph showing the expression of miR-320 by RT-PCR analysis after transfection of AC-16 cells either LNA-precursor miR-320 (LNA-miR-320) to knock down miR-320 expression or scrambled sequence (Scrambled) as the control in both study groups. Data are mean ± SEM and expressed as relative DCT. (B–D) Representative western blots and quantitative scatterplot bar graphs showing the expression of IGF-1 (B), Bcl-2 (C), and cleaved caspase-3 (D) in both the study groups after treatment with LNA-mIR-320 or scrambled sequence. Data are represented as the ratio to total protein and are mean ± SEM. Each western blot analysis was repeated three independent times. (E) Quantitative scatterplot bar graphs showing caspase-3/7 activity after normalizing the cell numbers using CyQUANT assay. Data are represented as a.u. and are mean ± SEM. (F) Quantitative scatterplot bar graphs showing absolute number of cells by CyQUANT assay and represented as mean ± SEM. *p < 0.05, **p < 0.01, and ****p < 0.001.

Figures 4D and S4C) and reduced caspase-3/7 activity ($p = 0.0006$ versus LNA-Scr-treated HG cells; Figure 4E). Interestingly, knockdown of miR-320 in NG cultured cells did not show any additive increase in IGF-1 expression (Figure 4B) or caspase-3/7 activity (Figure 4B), although there was a marked upregulation of Bcl-2 ($p = 0.05$ versus LNA-Scr-treated NG cells; Figure 4C). Inhibition of miR-320 did not have any effect of cell numbers (Figure 4F).

Prevention of miR-320a upregulation *in vivo* partially restored cardiac function in db/db mice

Finally, to determine if *in vitro* results are translatable *in vivo*, 24-week-old db/db mice were randomized to receive once a week intraperitoneal (i.p.) injection of LNA-scrambled (Scr) control or LNA-premiR-320 (10 mg/kg). Age-matched db/+ mice received only i.p. injection of LNA-Scr. Samples were collected at 8 weeks after the initiation of treatment. RT-PCR analysis showed no changes in the upregulated miR-320 expression in the diabetic heart treated with LNA-Scr ($p = 0.0195$ versus db/+ + LNA-Scr; Figure 5A). Interestingly, we observed a persistent decrease in the expression of miR-320 in db/db mice treated with LNA-premiR-320 ($p = 0.0410$ versus db/db + LNA-Scr; Figure 5A). The functional effect of the treatment was confirmed by preserved IGF-1 ($p = 0.0423$ versus db/db + LNA-Scr; Figures 5B and S5A) and Bcl-2 ($p = 0.0.0410$ versus db/db+ LNA-Scr; Figures 5C and S5B), and prevention of cleaved caspase-3 upregulation ($p = 0.0476$ versus db/db+ LNA-Scr; Figures 5D and S5C) in the LNA-premiR-320-treated db/db mice.

The histological analysis supported the molecular analysis data showing a significant increase in TUNEL-positive nuclei in the db/db heart treated with Scr (0.6193% ± 0.05339% in db/+ + LNA-Scr versus 1.909% ± 0.1583% in db/db + LNA-Scr, $p = 0.0001$; Figures 5E and S6), which was reduced following treatment with LNA-premiR-320 (1.333% ± 0.04138%, $p = 0.0004$ versus db/db + LNA-Scr; Figures 5E and S6). Vascular density analysis showed a significant improvement in the capillary density following miR-320 knockdown (1,223 ± 281/mm^2 in db/db + LNA-premiR-320 versus

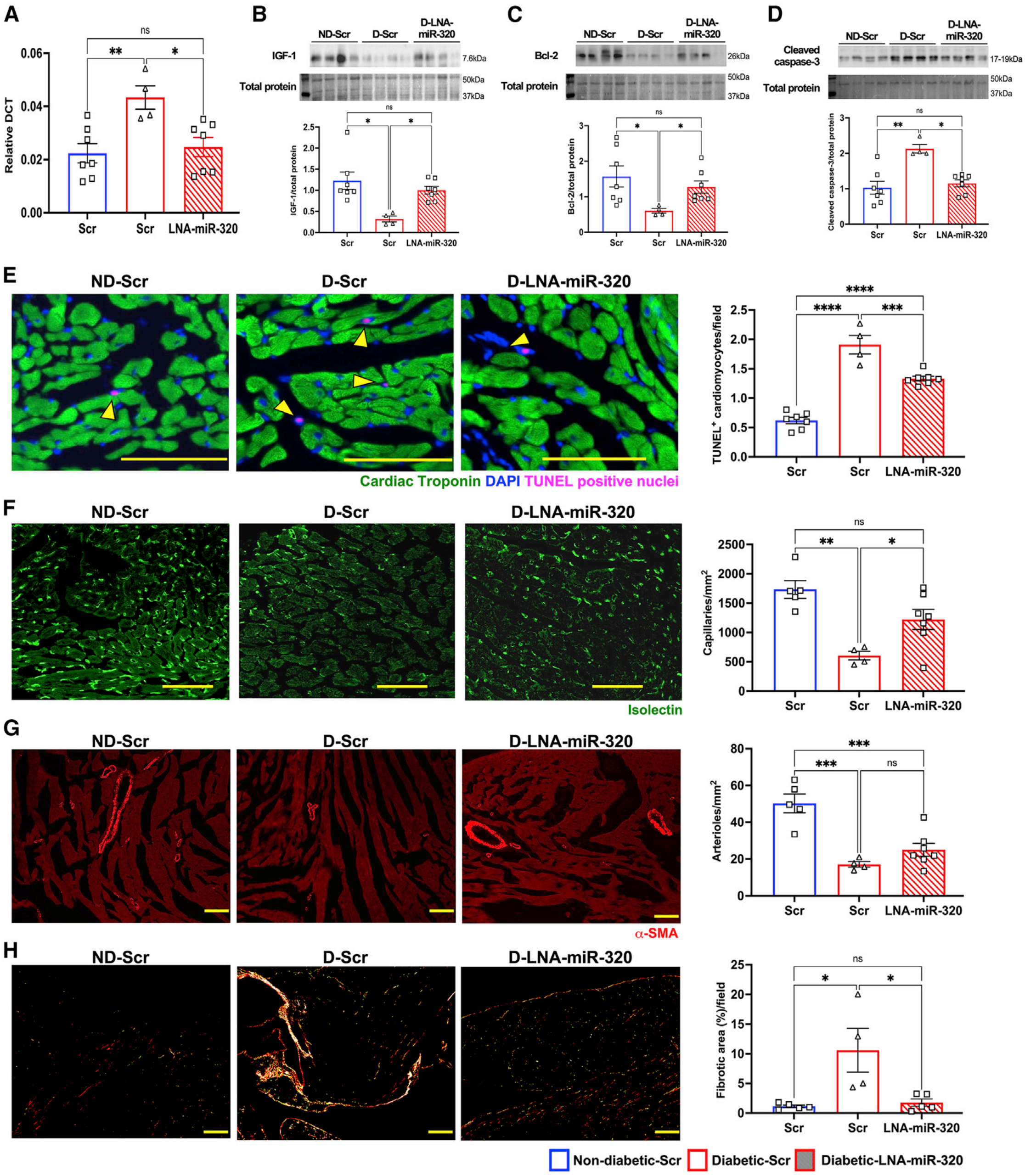

Figure 5. Therapeutic inhibition of miR-320 prevented adverse cardiac remodeling in type 2 diabetic db/db mice

(A) Quantitative scatterplot bar graph showing the expression of miR-320 expression by RT-PCR analysis after treatment of type 2 diabetic (db/db) mice with either LNA-precursor miR-320 (LNA-miR-320) to knock down miR-320 expression or scrambled sequence (Scrambled) as the control. Lean db/+ mice were only injected with a scrambled sequence. Data are mean ± SEM and expressed as relative DCT. (B–D) Representative western blots and quantitative scatterplot bar graphs showing the expression of IGF-1 (B), Bcl-2 (C), and cleaved caspase-3 (D) in all the study groups after treatment with LNA-mIR-320 or scrambled sequence. Data are represented as the ratio to total protein and are mean ± SEM. Each western blot analysis was repeated three independent times. (E) Representative fluorescent microscopy images and the quantitative scatterplot bar graphs showing the number of TUNEL positive cardiomyocytes (arrowhead) per field. Data are mean ± SEM. (F and G) Representative confocal microscopy images and the quantitative scatterplot bar graphs show the capillaries (F, green) and arterioles (G, red) among the study groups. Data are represented as a number of capillaries (F) and arterioles (G) per mm^2 and are mean ± SEM. (H) Representative microscopy images captured with polarized lens and the quantitative scatterplot bar graphs showing the percentage of fibrotic area per field among the study groups. Data are mean ± SEM. Five random images were taken at 200× magnification from each section, and three sections were used for each sample. Scale bars, 100 μm. *$p < 0.05$ and **$p < 0.01$. n = at least 4 animals in each group.

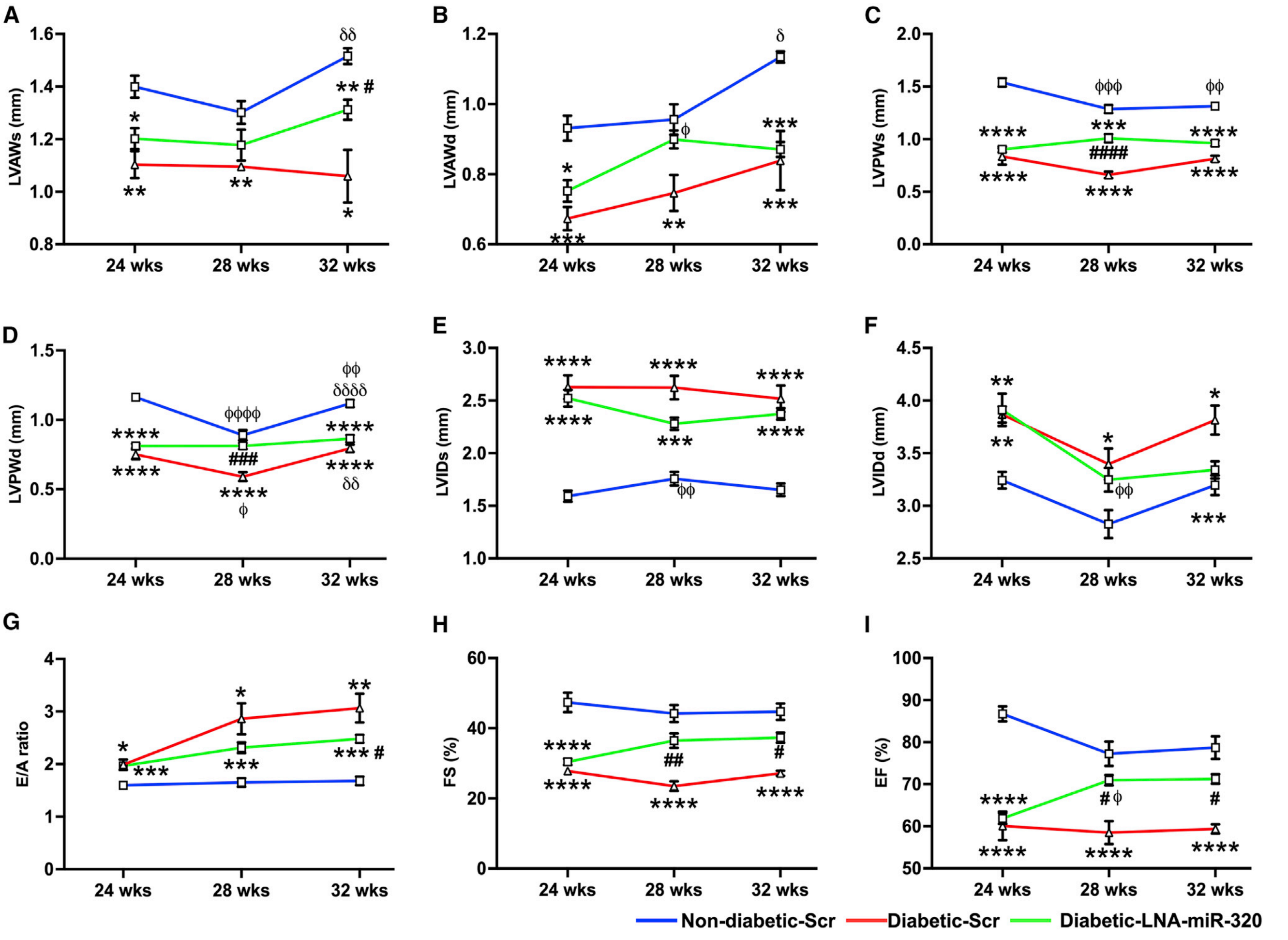

Figure 6. Therapeutic inhibition of miR-320 improved cardiac function

(A–I) Quantitative line graphs showing cardiac functions measured by echocardiography in all the study groups. Data are mean ± SEM. LVAWs, left ventricular anterior wall during systole; LVAWd, LVAW during diastole; LVPWs, LV posterior wall during systole; LVPWs, LVPW wall during diastole; LVIDs, LV internal diameter during systole; LVIDd, LVID during diastole; FS, fractional shortening; EF, ejection fraction. *$p < 0.05$, **$p < 0.01$, ***$p < 0.001$, and ****$p < 0.0001$ versus non-diabetic (ND) scrambled sequence (Scr)-treated group of the corresponding age; #$p < 0.05$ and ##$p < 0.01$ versus diabetic (D) scrambled (Scr)-treated group; φ$p < 0.05$, φφ$p<0.01$, φφφ$p<0.001$, and φφφφ$p<0.0001$ versus 24 weeks (wks) age time point; δδ$p<0.01$ and δδδδ$p<0.0001$ versus 28 weeks time point. n = 7 animals in each group.

454 ± 146/mm^2 in db/db + LNA-Scr, p = 0.04; Figures 5F and S7). While the density of small arterioles (<50 μm) showed a trend toward increase following the miR-320 knockdown, this was not significant (25 ± 9/mm^2 in db/db + LNA-premiR-320 versus 17 ± 3/mm^2 in db/db + LNA-Scr, p = 0.37; Figures 5G and S8). Furthermore, picrosirius red staining showed a marked reduction in structural remodeling of the diabetic heart as demonstrated by reduced interstitial fibrosis following treatment with LNA-premiR-320 (p = 0.02 versus db/bd + LNA-Scr-treated group; Figure 5H). Altogether, this improved both systolic and diastolic function in the db/db mice (Figure 6).

DISCUSSION

Our results have demonstrated a causal effect of miR-320 on increased apoptosis in the diabetic heart. Furthermore, we also showed that therapeutic knockdown of miR-320 in db/db mice prevented the downregulation of IGF-1, the direct target for miR-320, thereby preserving the downstream anti-apoptotic Bcl-2 signaling cascade to reduce apoptotic cell death and hence fibrotic remodeling. Overall, these resulted in improving cardiac function.

Wang et al. showed a positive correlation between miR-320 expression and HG exposure using neonatal cardiomyocytes. They also demonstrated that miR-320 released from HG-cultured cardiomyocytes induced endothelial cells dysfunction *in vitro*.[22] While these seminal pieces of evidence demonstrated upregulation of miR-320 in response to HG,[25–27] our finding is important because, using myocardial tissue samples collected at different time points, we were able to demonstrate that miR-320 upregulation occurs at the late stage in the diabetic heart. This is crucial for designing the therapeutic strategy for knockdown or

inhibiting miR-320 activity. A recent study showed that miR-320 could induce insulin resistance, affecting glucose transporter-4 (GLUT-4) in the cardiomyocytes to suppress glucose uptake.[26] Interestingly, our recent study showed marked downregulation of GLUT-4 in the late stage of diabetes, leading to reduced glucose uptake in the db/db mouse heart.[28] Although we did not measure the direct effect of miR-320 on glucose uptake, based on our earlier finding we speculate that upregulated miR-320 affected GLUT-4 expression, which reduced glucose uptake in the diabetic heart.

Cardiomyocytes are highly susceptible to apoptosis in diabetic conditions.[20] Several mechanisms have been postulated as the reason behind the increased apoptosis of diabetic cardiomyocytes. Among these, miRNAs are the key molecular mediators of cardiomyocyte apoptosis.[29] Tian et al. demonstrated a crucial role for miR-320 in inducing cardiomyocyte apoptosis in a mouse model of myocardial ischemia-reperfusion injury.[30] In another study, Cao and Chai demonstrated that reduced ischemia-reperfusion induced apoptosis following prevention of miR-320 upregulation by pharmacological preconditioning with morphine.[31] IGF-1, the target gene of miR-320, is a major survival growth factor that exhibits anti-apoptotic effects in the heart.[32] IGF-1 inhibits cardiomyocyte apoptosis by activating the downstream Bcl-2, which attenuates the induction of pro-apoptotic Bax and caspase-3. Our study showed a negative correlation between miR-320 and IGF-1 in the diabetic heart (data not shown) and, importantly, that this was associated with apoptotic cell death. Our *in vitro* studies further confirmed the causal role of miR-320 on IGF-1 downregulation and cardiomyocyte apoptosis, where upregulation of miR-320 preceded the downregulation of IGF-1, which in turn preceded cardiomyocyte apoptosis. Importantly, therapeutic knockdown of miR-320 stopped these chains of events, thereby suggesting a critical role of miR-320 in diabetic cardiomyocytes.

Type 2 diabetes is associated with a more than 2-fold increase in the development of both diastolic and systolic dysfunction.[33–35] Increased cardiomyocyte apoptosis is one of the major contributors to the development of cardiac dysfunction.[36] Loss of cardiomyocytes due to apoptosis results in fibrotic scar tissue formation, which, along with cardiac hypertrophy, makes the left ventricle stiff, a hallmark of diastolic dysfunction. The progression of the disease leads to systolic dysfunction.[37] This was supported by evidence from recent studies demonstrating a direct correlation between apoptosis and ejection fraction in patients with myocardial infarction.[38,39] In another study, reduction of apoptosis in a rat model of pressure overload markedly improved left ventricular fractional shortening.[40]

Interestingly, in our study, upregulation of miR-320 was observed only after the development of systolic dysfunction, suggesting that miR-320 does not initiate systolic dysfunction in the diabetic heart. However, increased apoptosis and fibrosis as a consequence of miR-320 upregulation is likely to have aggravated further deterioration of cardiac function, although whether miR-320 has a direct effect on fibrosis is not known. Furthermore, our study findings are contrary to a recent study that demonstrated upregulation of miR-320 expression before the development of systolic dysfunction in high fat diet-induced diabetic mice.[41] While the reason for the discrepancy in the results between the two studies is unknown, it is likely due to the difference in the model and underlying pathology to induce diabetes.

Our previous study identified the development of vascular rarefaction from 20 weeks of age in db/db mice.[19] In another study, we showed concurrent cardiac and vascular dysfunction development as a direct result of diabetes.[42] In the current study, miR-320 knockdown partially restored the lost capillaries in the diabetic heart. While miR-320 is predominantly expressed in cardiomyocytes, studies have demonstrated minimal expression of miR-320 in myocardial microvascular endothelial cells, where they are predicted to target angiogenic genes, such as fetal liver kinase-1, vascular endothelial growth factor-c, and fibroblast growth factors.[22] We also confirmed expression of miR-320 in endothelial cells, although there was no effect with HG. Previous study showed that miR-320 encapsulated in exosomes are released from cardiomyocytes and taken up by the microvascular endothelial cells to produce an anti-angiogenic effect.[43] This could have been the case in our study. Due to the focus of this study on cardiomyocytes, we did not explore in detail the underlying mechanisms for vascular improvement. Future mechanistic studies to determine the underlying pathways will be beneficial.

In conclusion, our study identified miR-320 as the late-responding miRNA in the diabetic heart. Although miR-320 do not initiate cardiac systolic or diastolic dysfunction, increased apoptosis and fibrosis due to miR-320 upregulation aggravated further deterioration of cardiac function. Importantly, therapeutic knockdown of miR-320 both *in vitro* and *in vivo* partially reversed diabetes-induced cardiac dysfunction. While AC16 cells are valuable as a model of *in vitro* cardiomyocytes due to the proliferative nature of these cells, we acknowledge that there may be a few differences in the metabolism compared with the primary cardiomyocytes.

Our approach to therapeutical knockdown of miR-320 *in vivo* is clinically relevant as we initiated the treatment after the onset of cardiac dysfunction. In clinical practice, most patients are currently diagnosed after developing cardiac dysfunction. Therefore, the ability to partially restore the deteriorated cardiac function through inhibition of apoptosis and fibrosis and restoration of microvasculatures in the diabetic heart further emphasizes the pathological role of miR-320 in the induction of cardiac dysfunction at the later stage of the disease. Hence, therapies targeting miR-320 are highly promising for the treatment of NiDHD.

MATERIAL AND METHODS

Ethics

The human myocardium study was approved by the Health and Disability Ethics Committee of New Zealand and the Human Ethics Committee at the University of Otago, Dunedin, New Zealand (approval nos. LRS/12/01/001 and HDEC#16/NTB/219). All the patients provided written consent for the collection and use of samples in this study. Collection and use of the human samples conformed to

Table 1. Patient characteristics

Sample ID	Age (years)	Sex	Diabetes duration (years)	Hypertension	Glucose level (Mmol/L)	HbA1c (mmol/ mol)	Medication
96	51	M	N/A	No	7.2	47	No
112	72	M	N/A	No	6.5	38.8	No
114	55	M	N/A	Yes	6.0	35.3	metoprolol and atorvastatin
145	77	M	N/A	Yes	N/A	N/A	amlodipine, metoprolol, and simvastatin
156	75	M	N/A	Yes	N/A	N/A	cilazapril, metoprolol, and simvastatin
167	66	M	N/A	Yes	5.5	35	metoprolol and atorvastatin
118	68	M	N/A	Yes	N/A	N/A	metoprolol and atorvastatin
189	70	M	N/A	No	5.4	39	atorvastatin
193	52	M	N/A	Yes	5.4	34	metoprolol and atorvastatin
196	60	M	N/A	Yes	5.5	35	metoprolol
202	76	M	N/A	Yes	6.4	38.1	amlodipine, metoprolol, and atorvastatin
211	68	M	N/A	Yes	4.4	34	metoprolol and simvastatin
197	75	M	13	Yes	8.5	52.6	bisoprolol, atorvastatin, metformin, and glipizide
335	61	M	10	Yes	12	49	amlodipine, cilazapril, metformin, hydrochlorothiazide, and glipizide
340	76	M	11	Yes	10	53	cilazapril, nadolol, simvastatin, and glipizide
438	56	M	8	Yes	13.1	57	metoprolol, glipizide, and atorvastatin
439	72	M	13	Yes	6.4	61	cilazapril, metoprolol, glipizide, and atorvastatin
521	71	M	14	Yes	14	54	cilazapril, metoprolol, glipizide, and atorvastatin
543	55	M	9	Yes	9.3	56	amlodipine, bisoprolol, insulin, and atorvastatin
630	68	M	14	Yes	8.6	68	quinapril, carvedilol, glipizide, atorvastatin, and aspirin
632	72	M	16	Yes	13.8	65	cilazapril, metoprolol, metformin, aspirin, and atorvastatin
633	61	M	9	Yes	7	50	quinapril, carvedolol, metformin, atorvastatin, and aspirin
642	67	M	12	Yes	11.2	53	metformin, glipizide, aspirin, simvastatin, and bisoprolol
715	57	M	10	Yes	13	50	amlodipine, cilazapril, metoprolol, glipizide, aspirin, and atorvastatin

the Declaration of Helsinki. All animal experiments were approved by the Animal Ethics Committee (approval no. AUP-18-205), University of Otago, New Zealand.

Experimental models and sample collection

Human myocardial tissue collection

RAA biopsies were collected from IHD patients with no history of diabetes (non-diabetic-IHD; ND-IHD, n = 12) and with a history of diabetes (diabetic-IHD; D-IHD, n = 12), who underwent on-pump coronary artery bypass graft surgery at Dunedin hospital, New Zealand (Table 1). In addition, to determine if ischemia alone had any effect of ischemia on miR-320, myocardial tissue samples collected from cadavers without any known history of ischemia or diabetes (ND-NIHD, n = 11) served as healthy controls. Myocardial samples were used from cadavers as we did not have access to the RAA. Moreover, our previous study showed no difference in the expression of miRNA between RAA and myocardium.[44]

Animal model of type 2 diabetes

In-house bred obese C57BL/KsJ-lepr^{db}/lepr^{db} (db/db) and age-matched lean control heterozygotes (db/+) mice (both male and female sex) were used as the model of type 2 diabetes and age-matched controls, respectively.[45] Animals were housed at the optimal room temperature of 21°C ± 1°C under a 12:12-h light-dark cycle. All the animals had access to water and a standard chow diet ad libitum. Our previous study showed the development of diastolic dysfunction in db/db mice from 20 weeks of age, which evolved into systolic dysfunction by 24 weeks.[46] To determine the expression pattern of miR-320 and its target protein IGF-1, myocardial samples were collected under terminal anesthesia (2,2,2-tribromoethanol,

640 mg/kg i.p. injection) from both db/db and db/+ mice at 8, 12, 16, 20, 24, 28, and 32 weeks of age.

Human ventricular cardiomyocyte (AC-16 cells) culture and HG exposure

AC-16 cells were purchased from Davidson Laboratory at the University of Colombia, New York.[47] Cells were maintained in Dulbecco's modified Eagle medium: Nutrient Mixture F12 (DMEM/F12) (Life Technologies, New Zealand) with 12.5% fetal bovine serum (FBS) (Thermo Fisher Scientific, New Zealand) and 1% penicillin-streptomycin solution (Thermo Fisher Scientific, New Zealand) at 37°C with a gas mixture of 5% CO_2 with 95% air. Cells were cultured in DMEM with 5.5 mM glucose (NG, Thermo Fisher Scientific, New Zealand) for at least four passages before exposing them to high D-glucose (HG; 30 mM, Sigma-Merck, New Zealand) for 24 to 144 h to simulate *in vitro* diabetic condition. As the osmotic control, cells cultured in NG were supplemented with D-mannitol (30 mM, Sigma-Merck, New Zealand). Cells were seeded at 2.5×10^4 cells per well in 12-well plates for RNA extraction, 4×10^3 cells per well in 96-well plates for caspase-3/7 activity assay, and at 3×10^5 cells per 60-mm dish for protein extraction. Samples for RNA and protein extraction were collected at 24, 48, 72, 96, 120, and 144 h following exposure to HG. Caspase-3/7 activity was measured at 144 h.

Experiments using human umbilical vein endothelial cells and human cardiac fibroblasts are detailed in the online supplemental information.

Therapeutic modulation of miR-320

In vitro *knockdown of miR-320*

To determine if *in vitro* knockdown of miR-320 reverse HG-induced apoptosis in cardiomyocytes, AC-16 cells (3×10^6 cells/well in a 6-well plate) were exposed to 72 h of HG. Following this, cells were transfected with LNA-premiR-320 or LNA-Scr (both from QIAGEN, Germany) using Lipofectamine RNAiMAX (Thermo Fisher Scientific, USA). In brief, 5 pmol LNA-premiR-320 or LNA-Scr was mixed with 5 μL Lipofectamine RNAiMax Transfection Reagent (Thermo Fisher Scientific, USA) in 200 μL DMEM without serum (Thermo Fisher Scientific, USA) and incubated for 15 min at room temperature. Then, the complex was added to the cells and swirled carefully for even distribution over the entire plate surface. We used LNA against precursor miR-320 to knockdown mature miR-320 expression rather than inhibiting the activity of mature miR-320 using anti-miR. At 120 h of HG treatment, the cell lysates were collected for RNA and protein extraction along with the measurement of caspase-3/7 activity.

In vivo *knockdown of miR-320*

Following baseline echocardiography (GE Vivid E9) measurement, 24-week-old db/db mice (n = 7 in each group) were randomized to receive once a week i.p. injection of LNA- Scr control or LNA-premiR-320 (10 mg/kg, both from QIAGEN, Germany). Age-matched db/+ mice received only i.p. injection of LNA-Scr, as they did not show any difference in miR-320 expression.[48] Cardiac function was measured every 4 weeks using a Vivid E9 cardiovascular ultrasound system (GE Vingmed Ultrasound, Horten, Norway). Left ventricular wall thickness, systolic function (internal diameters, ejection fraction, fractional shortening) and diastolic dysfunction (E/A ratio, deceleration time [DecT]) were assessed as described previously by us.[18,28,49] Animals were initially planned to receive 8 weeks of consecutive injections. However, the injection had to be terminated after 4 weeks because of a nationwide lockdown due to the COVID-19 pandemic (March 2020). In addition, previous studies have shown the effects of a single dose of LNA injection on miRNA inhibition. Therefore, we decided to continue monitoring the animals until the desired study period. At the end of 8 weeks after the first injection, following echocardiography measurement, under terminal anesthesia, the hearts were quickly removed, washed in phosphate-buffered saline, and dissected into three sections from apex to base for molecular (RNA and protein) and histological analysis.

Expression analysis

Total RNA isolation and real-time qRT-PCR analysis

Total RNA from heart tissue (human and mouse) and AC-16 cells were isolated using QIAzol reagent as per the manufacturer's instructions.[50] Twenty nanograms of total RNA was reverse transcribed using miR-320-specific stem-loop structure reverse transcription primer (Thermo Fisher Scientific, New Zealand), followed by amplification. U6 small-nuclear RNA was used as the internal control. Relative expression was expressed as $2^{-\Delta CT}$.

Western blot analysis

Cardiac tissue (human and mouse) and AC-16 cells were homogenized in ice-cold RIPA lysis buffer supplemented with a 1% protease inhibitor cocktail (Sigma-Aldrich, USA).[51] For human tissues, due to the small size (5 mg) of samples in the ND-NIHD group, protein expression was restricted to only ND-IHD and D-IHD groups. Total protein was quantified using the Bradford protein assay (BioRad, New Zealand). Twenty or 40 μg of protein were resolved by 15% SDS-PAGE and transferred to a polyvinylidene fluoride membrane. After blocking and confirming the successful transfer of proteins using Ponceau staining, membranes were probed with primary antibodies against IGF-1 (1:1,000 dilution, BioRad), B cell lymphoma-2 (Bcl-2; 1:1,000, Cell Signaling Technology), and cleaved caspase-3 (1:500 dilution, Cell Signaling Technology) overnight at 4°C. Goat anti-rabbit secondary antibody (1:10,000 dilution, Abcam) was used for detection. The density of bands was analyzed using ImageJ (NIH, USA) software and normalized to total protein as detected using Ponceau staining.

Functional analysis

Caspase-3/7 activity assay

At the end of the treatment period, cells seeded in the 96-well plate were washed with PBS and supplemented with a mixture consisting of 25 μL of caspase reagent (Promega, USA) and 25 μL of fresh medium. The reagents were gently mixed on a plate shaker at 250 rpm for 1 min and incubated at room temperature for 30 min. Following incubation, luminescence was measured using a plate reader (SpectraMax i3x; Molecular Devices, USA). Next, 50 μL of 2× concentrated CyQUANT

reagent (Thermo Fisher Scientific, New Zealand) was added to each well. The plate was then incubated at room temperature for 10 min in the dark, and fluorescence was measured using the plate reader (excitation at 480 nm and emission at 520 nm, SpectraMax i3x; Molecular Devices, USA). Caspase activity (relative luminescent units) was then normalized to the cell numbers measured using the CyQUANT reagent (relative fluorescence units) and represented as caspase activity/cell number.

Microvascular analysis

For histological analysis, tissue sections collected from mouse heart were fixed overnight in freshly prepared 4% paraformaldehyde, followed by cryoprotection in 30% sucrose in PBS solution. The sections were then embedded in Tissue-Tek OCT compound and frozen by placing them on cold isopentane/2-methylbutane. Frozen sections were stored at -80°C until cryosectioned. Seven-micron-thick myocardial cryosections were then probed with biotin-conjugated Isolectin-B4 (1:200 dilution, Vector laboratories, USA) and anti-α-smooth muscle actin conjugated with Cy3 (1:800 dilution, Sigma-Aldrich, USA) to detect endothelial cells (to determine the capillary density) and smooth muscle cells (to determine the arteriole density), respectively. DAPI (1:1,000 dilution, Santa Cruz Biotechnologies, USA) was used to stain the nuclei. Images were captured at 200× magnification using a confocal microscope (Nikon A1). The density of capillaries was expressed as the mean number of isolectin$^+$ cells/mm^2 of cardiac tissue. The density of arterioles (<50 μm lumen size) was expressed as the mean number of αSMA$^+$isolectin$^+$ cells/mm^2 of cardiac tissue.

In situ *detection of apoptosis*

Apoptotic cardiomyocytes were detected in 7-μm-thick myocardial cryosections using terminal deoxynucleotidyl transferase dUTP nick end labeling (TUNEL) staining following the manufacturer's protocol (Thermo Fisher Scientific, New Zealand). Following this, sections were probed with cardiac troponin mouse monoclonal antibody (1:100 dilution, Novus Biologicals, USA) to label cardiomyocytes and counterstained with Alexa Fluro 633 goat-anti-mouse secondary antibody (1:1,000 dilution, Thermo Fisher Scientific, New Zealand). Images were captured at 200× magnification using an Olympus B×51 fluorescence microscope. Data are expressed as the percentage of TUNEL-positive cardiomyocytes in the section.

Picrosirius red staining to measure cardiac fibrosis

Cardiac fibrosis was assessed by picrosirius red staining as described earlier.[52] In brief, 7-μm-thick myocardial cryosections were fixed in acetone and stained with 1% picrosirius red for 1 h, washed in acidified H_2O, followed by dehydration in an ascending ethanol concentration. Following staining, images were captured using a Nikon bright-field microscope fitted with a polarized lens (Nikon Ti2-E). The fibrotic area was normalized to the total tissue area and expressed as a percentage of fibrotic tissue. All data were analyzed using ImageJ software (NIH).

Statistical analysis

All statistical analyses were performed using GraphPad Prism Software. Data are expressed as mean ± SEM. Shapiro-Wilk test was used to test the normality of data distribution. A one-way ANOVA, followed by a non-parametric Kruskal-Wallis test, was used to determine the statistical difference between (1) the miRNA expression collected from different human RAA tissues and (2) the miRNA, target protein and apoptotic marker expression, and hemodynamic parameters for LNA-treated mouse heart samples. A two-way ANOVA, followed by a Tukey's multiple comparisons test, was used to assess the statistical difference in miRNA expression, target protein expression, and caspase-3/7 activity between normal and HG-cultured AC-16 cells at baseline and after LNA treatment. A Student's t test was used to determine the statistical difference in target protein and apoptotic markers between diabetic and non-diabetic RAA tissue. A p value <0.05 was predetermined as the level of significance for all statistical analyses.

DATA AVAILABILITY

All data associated with this study are presented in the paper and supplemental information.

SUPPLEMENTAL INFORMATION

Supplemental information can be found online at https://doi.org/10.1016/j.omtn.2022.07.007.

ACKNOWLEDGMENTS

We thank the staff nurses at the surgical theater in helping with human sample collection. We also thank the animal welfare technicians for their help in daily monitoring and care of our experimental animals. We thank Mr Andew McNaughton and Mr Rob Wooley of Otago Micro and Nanoscale imaging for help in acquiring images. This study was supported by the PhD research funding from the Department of Physiology, University of Otago.

AUTHOR CONTRIBUTIONS

All authors contributed to the interpretation of data and reviewing of the manuscript. N.G. performed all the *in vitro* and *in vivo* experimental work, data analysis, and drafted the manuscript; S.F. performed immunohistochemical analysis of vascular density; I.v.H. maintained the database for human tissue samples; G.J. provided non-ischemic cadaveric samples; S.C. and M.J.A.W. provided access to the clinical data; R.S., D.P., and P.D. acquired the human tissue samples; D.O.S. provided expert advice on animal studies; A.C. was the co-supervisor for N.G. and critically reviewed the manuscript; R.K. conceived, designed, and supervised the study, and wrote the final version of the manuscript.

DECLARATION OF INTERESTS

The authors declare they have no conflict of interest.

REFERENCES

1. Anderson, J.L., Adams, C.D., Antman, E.M., Bridges, C.R., Califf, R.M., Casey, D.E., Chavey, W.E., Fesmire, F.M., Hochman, J.S., Levin, T.N., et al. (2007). ACC/AHA 2007 guidelines for the management of patients with unstable angina/non–ST-elevation myocardial infarction: a report of the American College of Cardiology/American heart association task force on practice guidelines (writing Committee to revise the 2002 guidelines for the management of patients with unstable Angina/non–ST-elevation myocardial infarction) developed in collaboration with the American

College of emergency physicians, the society for cardiovascular angiography and interventions, and the society of thoracic surgeons endorsed by the American association of cardiovascular and pulmonary rehabilitation and the society for academic emergency medicine. J. Am. Coll. Cardiol. *50*, e1–e157.

2. Bell, D.S.H. (2003). Heart failure. Diabetes Care *26*, 2433–2441.

3. From, A.M., Leibson, C.L., Bursi, F., Redfield, M.M., Weston, S.A., Jacobsen, S.J., Rodeheffer, R.J., and Roger, V.L. (2006). Diabetes in heart failure: prevalence and impact on outcome in the population. Am. J. Med. *119*, 591–599.

4. Kim, A.H., Jang, J.E., and Han, J. (2022). Current status on the therapeutic strategies for heart failure and diabetic cardiomyopathy. Biomed. Pharmacother. *145*, 112463. https://doi.org/10.1016/j.biopha.2021.112463.

5. Ghosh, N., and Katare, R. (2018). Molecular mechanism of diabetic cardiomyopathy and modulation of microRNA function by synthetic oligonucleotides. Cardiovasc. Diabetol. *17*, 43. https://doi.org/10.1186/s12933-018-0684-1.

6. Boudina, S., and Abel, E.D. (2010). Diabetic cardiomyopathy, causes and effects. Rev. Endocr. Metab. Disord. *11*, 31–39.

7. Cao, Y., Lin, S., and Li, X. (2015). Acute pulmonary edema as first clinical presentation in a patient with hypertrophic cardiomyopathy. Zhonghua Xin Xue Guan Bing Za Zhi *43*, 828.

8. Peters, S. (2016). Echocardiographic correlate of myocardial edema in complicated takotsubo cardiomyopathy. Int. J. Cardiol. *215*, 299–300.

9. Schenke-Layland, K., Stock, U.A., Nsair, A., Xie, J., Angelis, E., Fonseca, C.G., Larbig, R., Mahajan, A., Shivkumar, K., Fishbein, M.C., and MacLellan, W.R. (2009). Cardiomyopathy is associated with structural remodelling of heart valve extracellular matrix. Eur. Heart J. *30*, 2254–2265.

10. Gilbert, R.E., and Krum, H. (2015). Heart failure in diabetes: effects of anti-hyperglycaemic drug therapy. Lancet *385*, 2107–2117.

11. Li, H., Fan, J., Zhao, Y., Zhang, X., Dai, B., Zhan, J., Yin, Z., Nie, X., Fu, X.-D., Chen, C., and Wang, D.W. (2019). Nuclear miR-320 mediates diabetes-induced cardiac dysfunction by activating transcription of fatty acid metabolic genes to cause lipotoxicity in the heart. Circ. Res. *125*, 1106–1120.

12. Ghosh, N., and Katare, R. (2018). Molecular mechanism of diabetic cardiomyopathy and modulation of microRNA function by synthetic oligonucleotides. Cardiovasc. Diabetol. *17*, 43.

13. Bartel, D.P., and Chen, C.-Z. (2004). Micromanagers of gene expression: the potentially widespread influence of metazoan microRNAs. Nat. Rev. Genet. *5*, 396–400.

14. Diao, X., Shen, E., Wang, X., and Hu, B. (2011). Differentially expressed microRNAs and their target genes in the hearts of streptozotocin-induced diabetic mice. Mol. Med. Rep. *4*, 633–640.

15. Guo, R., and Nair, S. (2017). Role of MicroRNA in diabetic cardiomyopathy: from mechanism to intervention. Biochim. Biophys. Acta *1863*, 2070–2077.

16. Purvis, N., Kumari, S., Chandrasekera, D., Bellae Papannarao, J., Gandhi, S., van Hout, I., Coffey, S., Bunton, R., Sugunesegran, R., Parry, D., et al. (2021). Diabetes induces dysregulation of microRNAs associated with survival, proliferation and self-renewal in cardiac progenitor cells. Diabetologia *64*, 1422–1435. https://doi.org/10.1007/s00125-021-05405-7.

17. Lew, J.K.S., Pearson, J.T., Saw, E., Tsuchimochi, H., Wei, M., Ghosh, N., Du, C.K., Zhan, D.Y., Jin, M., Umetani, K., et al. (2020). Exercise regulates MicroRNAs to preserve coronary and cardiac function in the diabetic heart. Circ. Res. *127*, 1384–1400. https://doi.org/10.1161/CIRCRESAHA.120.317604.

18. Rawal, S., Nagesh, P.T., Coffey, S., Van Hout, I., Galvin, I.F., Bunton, R.W., Davis, P., Williams, M.J.A., and Katare, R. (2019). Early dysregulation of cardiac-specific microRNA-208a is linked to maladaptive cardiac remodelling in diabetic myocardium. Cardiovasc. Diabetol. *18*, 13. https://doi.org/10.1186/s12933-019-0814-4.

19. Rawal, S., Munasinghe, P.E., Shindikar, A., Paulin, J., Cameron, V., Manning, P., Williams, M.J.A., Jones, G.T., Bunton, R., Galvin, I., and Katare, R. (2017). Down-regulation of proangiogenic microRNA-126 and microRNA-132 are early modulators of diabetic cardiac microangiopathy. Cardiovasc. Res. *113*, 90–101. https://doi.org/10.1093/cvr/cvw235.

20. Booz, G.W., and Baker, K.M. (1995). Molecular signalling mechanisms controlling growth and function of cardiac fibroblasts. Cardiovasc. Res. *30*, 537–543.

21. Song, C.-L., Liu, B., Diao, H.-Y., Shi, Y.-F., Zhang, J.-C., Li, Y.-X., Liu, N., Yu, Y.-P., Wang, G., Wang, J.-P., and Li, Q. (2016). Down-regulation of microRNA-320 suppresses cardiomyocyte apoptosis and protects against myocardial ischemia and reperfusion injury by targeting IGF-1. Oncotarget *7*, 39740–39757.

22. Wang, X.H., Qian, R.Z., Zhang, W., Chen, S.F., Jin, H.M., and Hu, R.M. (2009). MicroRNA-320 expression in myocardial microvascular endothelial cells and its relationship with insulin-like growth factor-1 in type 2 diabetic rats. Clin. Exp. Pharmacol. Physiol. *36*, 181–188.

23. Wang, X.H., Qian, R.Z., Zhang, W., Chen, S.F., Jin, H.M., and Hu, R.M. (2009). MicroRNA-320 expression in myocardial microvascular endothelial cells and its relationship with insulin-like growth factor-1 in type 2 diabetic rats. Clin. Exp. Pharmacol. Physiol. *36*, 181–188. https://doi.org/10.1111/j.1440-1681.2008.05057.x.

24. Ola, M.S., Nawaz, M., and Ahsan, H. (2011). Role of Bcl-2 family proteins and caspases in the regulation of apoptosis. Mol. Cell. Biochem. *351*, 41–58.

25. Chakraborty, C., Doss, C.G., Bandyopadhyay, S., and Agoramoorthy, G. (2014). Influence of miRNA in insulin signaling pathway and insulin resistance: micro-molecules with a major role in type-2 diabetes. Wiley Interdiscip Rev RNA *5*, 697–712. https://doi.org/10.1002/wrna.1240.

26. Du, H., Zhao, Y., Yin, Z., Wang, D.W., and Chen, C. (2021). The role of miR-320 in glucose and lipid metabolism disorder-associated diseases. Int. J. Biol. Sci. *17*, 402–416. https://doi.org/10.7150/ijbs.53419.

27. Feng, B., and Chakrabarti, S. (2012). miR-320 regulates glucose-induced gene expression in diabetes. ISRN Endocrinol. *2012*, 549875. https://doi.org/10.5402/2012/549875.

28. Saw, E.L., Pearson, J.T., Schwenke, D.O., Munasinghe, P.E., Tsuchimochi, H., Rawal, S., Coffey, S., Davis, P., Bunton, R., Van Hout, I., et al. (2021). Activation of the cardiac non-neuronal cholinergic system prevents the development of diabetes-associated cardiovascular complications. Cardiovasc. Diabetol. *20*, 50. https://doi.org/10.1186/s12933-021-01231-8.

29. Skommer, J., Rana, I., Marques, F.Z., Zhu, W., Du, Z., and Charchar, F.J. (2014). Small molecules, big effects: the role of microRNAs in regulation of cardiomyocyte death. Cell Death Dis. *5*, e1325. https://doi.org/10.1038/cddis.2014.287.

30. Tian, Z.Q., Jiang, H., and Lu, Z.B. (2018). MiR-320 regulates cardiomyocyte apoptosis induced by ischemia-reperfusion injury by targeting AKIP1. Cell. Mol. Biol. Lett. *23*, 41. https://doi.org/10.1186/s11658-018-0105-1.

31. Cao, L., and Chai, S. (2020). miR3203p is involved in morphine preconditioning to protect rat cardiomyocytes from ischemia/reperfusion injury through targeting Akt3. Mol. Med. Rep. *22*, 1480–1488. https://doi.org/10.3892/mmr.2020.11190.

32. Baines, C.P., and Molkentin, J.D. (2005). STRESS signaling pathways that modulate cardiac myocyte apoptosis. J. Mol. Cell. Cardiol. *38*, 47–62. https://doi.org/10.1016/j.yjmcc.2004.11.004.

33. Kenny, H.C., and Abel, E.D. (2019). Heart failure in type 2 diabetes mellitus. Circ. Res. *124*, 121–141. https://doi.org/10.1161/CIRCRESAHA.118.311371.

34. MacDonald, M.R., Petrie, M.C., Hawkins, N.M., Petrie, J.R., Fisher, M., McKelvie, R., Aguilar, D., Krum, H., and McMurray, J.J.V. (2008). Diabetes, left ventricular systolic dysfunction, and chronic heart failure. Eur. Heart J. *29*, 1224–1240. https://doi.org/10.1093/eurheartj/ehn156.

35. Bergerot, C., Davidsen, E.S., Amaz, C., Thibault, H., Altman, M., Bellaton, A., Moulin, P., Derumeaux, G., and Ernande, L. (2018). Diastolic function deterioration in type 2 diabetes mellitus: predictive factors over a 3-year follow-up. Eur. Heart J. Cardiovasc. Imag. *19*, 67–73. https://doi.org/10.1093/ehjci/jew331.

36. Narula, J., Haider, N., Arbustini, E., and Chandrashekhar, Y. (2006). Mechanisms of disease: apoptosis in heart failure–seeing hope in death. Nat. Clin. Pract. Cardiovasc. Med. *3*, 681–688. https://doi.org/10.1038/ncpcardio0710.

37. Salvatore, T., Pafundi, P.C., Galiero, R., Albanese, G., Di Martino, A., Caturano, A., Vetrano, E., Rinaldi, L., and Sasso, F.C. (2021). The diabetic cardiomyopathy: the contributing pathophysiological mechanisms. Front. Med. *8*, 695792. https://doi.org/10.3389/fmed.2021.695792.

38. Teringova, E., Kozel, M., Knot, J., Kocka, V., Benesova, K., and Tousek, P. (2018). Relationship between TRAIL and left ventricular ejection fraction in patients with ST-elevation myocardial infarction treated with primary percutaneous coronary intervention. Biomed Res. Int. *2018*, 3709084.

39. Lew, J.K.-S., Pearson, J.T., Saw, E., Tsuchimochi, H., Wei, M., Ghosh, N., Du, C.-K., Zhan, D.-Y., Jin, M., Umetani, K., et al. (2020). Exercise regulates microRNAs to preserve coronary and cardiac function in the diabetic heart. Circ. Res. *127*, 1384–1400.

40. Gelpi, R.J., Park, M., Gao, S., Dhar, S., Vatner, D.E., and Vatner, S.F. (2011). Apoptosis in severe, compensated pressure overload predominates in nonmyocytes and is related to the hypertrophy but not function. Am. J. Physiol. Heart Circ. Physiol. *300*, H1062–H1068.

41. Li, H., Fan, J., Zhao, Y., Zhang, X., Dai, B., Zhan, J., Yin, Z., Nie, X., Fu, X.-D., Chen, C., and Wang, D.W. (2019). Nuclear miR-320 mediates diabetes-induced cardiac dysfunction by activating transcription of fatty acid metabolic genes to cause lipotoxicity in the heart. Circ. Res. *125*, 1106–1120. https://doi.org/10.1161/CIRCRESAHA.119.314898.

42. Katare, R., Pearson, J.T., Lew, J.K.S., Wei, M., Tsuchimouchi, H., Du, C.K., Zhan, D.Y., Umetani, K., Shirai, M., and Schwenke, D.O. (2018). Progressive decrease in coronary vascular function associated with type 2 diabetic heart disease. Front. Physiol. *9*, 696. https://doi.org/10.3389/fphys.2018.00696.

43. Wang, X., Huang, W., Liu, G., Cai, W., Millard, R.W., Wang, Y., Chang, J., Peng, T., and Fan, G.C. (2014). Cardiomyocytes mediate anti-angiogenesis in type 2 diabetic rats through the exosomal transfer of miR-320 into endothelial cells. J. Mol. Cell. Cardiol. *74*, 139–150. https://doi.org/10.1016/j.yjmcc.2014.05.001.

44. Rawal, S., Ram, T.P., Coffey, S., Williams, M.J.A., Saxena, P., Bunton, R.W., Galvin, I.F., and Katare, R. (2016). Differential expression pattern of cardiovascular microRNAs in the human type-2 diabetic heart with normal ejection fraction. Int. J. Cardiol. *202*, 40–43. https://doi.org/10.1016/j.ijcard.2015.08.161.

45. Bugger, H., and Abel, E.D. (2009). Rodent models of diabetic cardiomyopathy. Dis. Model. Mech. *2*, 454–466.

46. Rawal, S., Nagesh, P.T., Coffey, S., Van Hout, I., Galvin, I.F., Bunton, R.W., Davis, P., Williams, M.J.A., and Katare, R. (2019). Early dysregulation of cardiac-specific microRNA-208a is linked to maladaptive cardiac remodelling in diabetic myocardium. Cardiovasc. Diabetol. *18*, 13.

47. Davidson, M.M., Nesti, C., Palenzuela, L., Walker, W.F., Hernandez, E., Protas, L., Hirano, M., and Isaac, N.D. (2005). Novel cell lines derived from adult human ventricular cardiomyocytes. J. Mol. Cell. Cardiol. *39*, 133–147.

48. Bernardo, B.C., Gao, X.-M., Tham, Y.K., Kiriazis, H., Winbanks, C.E., Ooi, J.Y.Y., Boey, E.J.H., Obad, S., Kauppinen, S., Gregorevic, P., et al. (2014). Silencing of miR-34a attenuates cardiac dysfunction in a setting of moderate, but not severe, hypertrophic cardiomyopathy. PLoS One *9*, e90337.

49. Rawal, S., Munasinghe, P.E., Nagesh, P.T., Lew, J.K.S., Jones, G.T., Williams, M.J.A., Davis, P., Bunton, D., Galvin, I.F., Manning, P., et al. (2017). Down-regulation of miR-15a/b accelerates fibrotic remodelling in the Type 2 diabetic human and mouse heart. Clin. Sci. *131*, 847–863. https://doi.org/10.1042/CS20160916.

50. Wong, L.L., Rademaker, M.T., Saw, E.L., Lew, K.S., Ellmers, L.J., Charles, C.J., Richards, A.M., and Wang, P. (2017). Identification of novel microRNAs in the sheep heart and their regulation in heart failure. Sci. Rep. *7*, 8250.

51. Gao, X.-F., Zhou, Y., Wang, D.-Y., Lew, K.-S., Richards, A.M., and Wang, P. (2015). Urocortin-2 suppression of p38-MAPK signaling as an additional mechanism for ischemic cardioprotection. Mol. Cell. Biochem. *398*, 135–146.

52. Rawal, S., Munasinghe, P.E., Nagesh, P.T., Lew, J.K.S., Jones, G.T., Williams, M.J.A., Davis, P., Bunton, D., Galvin, I.F., Manning, P., et al. (2017). Down-regulation of miR-15a/b accelerates fibrotic remodelling in the Type 2 diabetic human and mouse heart. Clin. Sci. *131*, 847–863.

OMTN, Volume *29*

Supplemental information

Therapeutic knockdown of miR-320 improves deteriorated cardiac function in a pre-clinical model of non-ischemic diabetic heart disease

Nilanjan Ghosh, Sonya Fenton, Isabelle van Hout, Gregory T. Jones, Sean Coffey, Michael J.A. Williams, Ramanen Sugunesegran, Dominic Parry, Philip Davis, Daryl O. Schwenke, Anirudha Chatterjee, and Rajesh Katare

Supplemental Material

Supplemental Methods

Human umbilical vein endothelial cells (HUVEC) culture and high glucose exposure

Human Umbilical Vein Endothelial Cells (HUVECs) were purchased from Lonza and cultured in EGM-2 complete media (Lonza) as per manufacturer's protocol and as described in detail in our previous study[1]. For experiments, cells (0.05 x 10^6/well in 24 well plate) were exposed to high D-glucose (HG, 30mM) or D-mannitol (NG, 30mM, used as osmotic control). Samples for RNA were collected at 24, 48, 72, 96, 120 and 144 hours following exposure to HG.

Human cardiac fibroblasts culture and high glucose exposure

The human primary cardiac fibroblasts were obtained by outgrowth from right atrial appendage (RAA) biopsy samples collected from patients undergoing coronary artery bypass graft surgery for our previous study[2,3] was used in this study. The frozen cells were plated in a T75 culture flask in RPMI 1640 medium (ThermoFisher, USA) supplemented with 10% fetal bovine serum (FBS) and antibiotics. For experiments, cells (3 x 10^5/well in 6 well plate) were exposed to high D-glucose (HG, 30mM) or D-mannitol (NG, 30mM, used as osmotic control). Samples for RNA were collected at 24, 48, 72, 96, 120 and 144 hours following exposure to HG.

References

1. Rawal, S., Munasinghe, P.E., Shindikar, A., Paulin, J., Cameron, V., Manning, P., Williams, M.J., Jones, G.T., Bunton, R., Galvin, I., and Katare, R. (2017). Down-regulation of proangiogenic microRNA-126 and microRNA-132 are early modulators of diabetic cardiac microangiopathy. Cardiovasc Res *113*, 90-101. 10.1093/cvr/cvw235.

2. Rawal, S., Munasinghe, P.E., Nagesh, P.T., Lew, J.K.S., Jones, G.T., Williams, M.J.A., Davis, P., Bunton, D., Galvin, I.F., Manning, P., Lamberts, R.R., et al. (2017). Down-regulation of miR-15a/b accelerates fibrotic remodelling in the Type 2 diabetic human and mouse heart. Clin Sci (Lond) *131*, 847-863. 10.1042/CS20160916.

3. S., Y.-G.A., A, Y., I, v.H., J, B.P., R, S., D, P., P, D., and Katare, R. (2022). Cardiac Progenitor Cells and Adipocyte Stem Cells from Same Patients Exhibit In Vitro Functional Differences. International Journal of molecular science *17*. 10.3390/ijms23105588.

Supplemental Figure 1

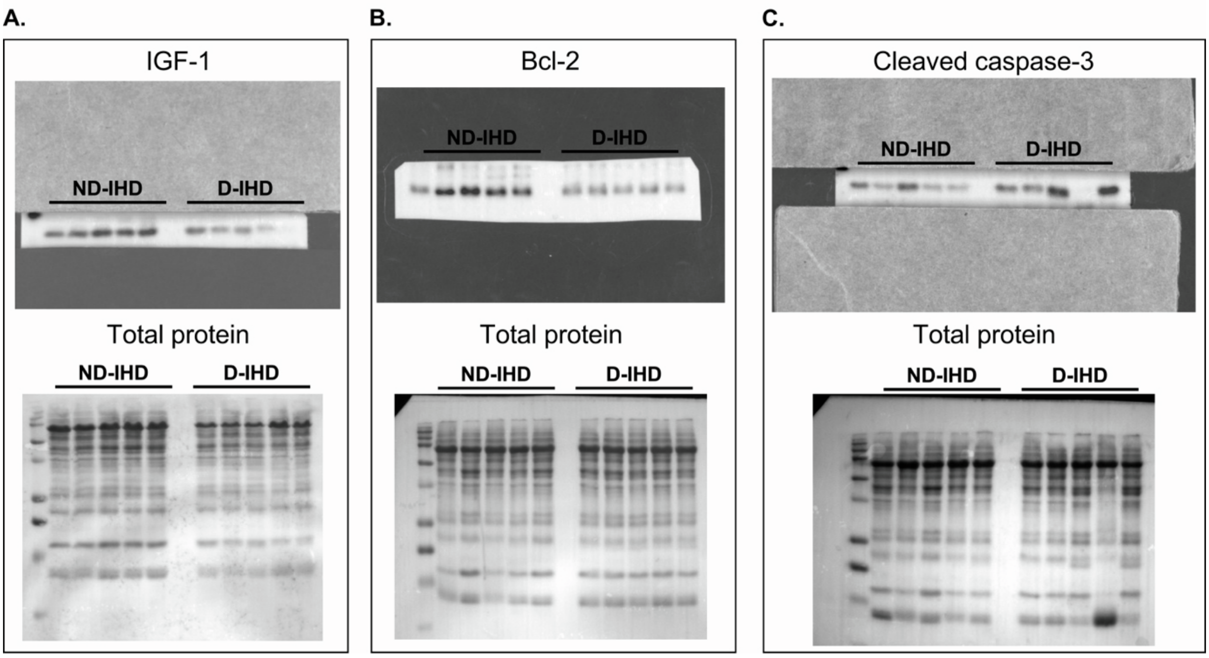

Supplemental Figure 1. Uncropped blots showing the expression of IGF-1 (**A, shown in Figure 1B**), Bcl-2 (**B, shown in Figure 1C**), and cleaved caspase-3 (CC3, **C, shown in Figure 1D**) in human RAA tissue.

Supplemental Figure 2

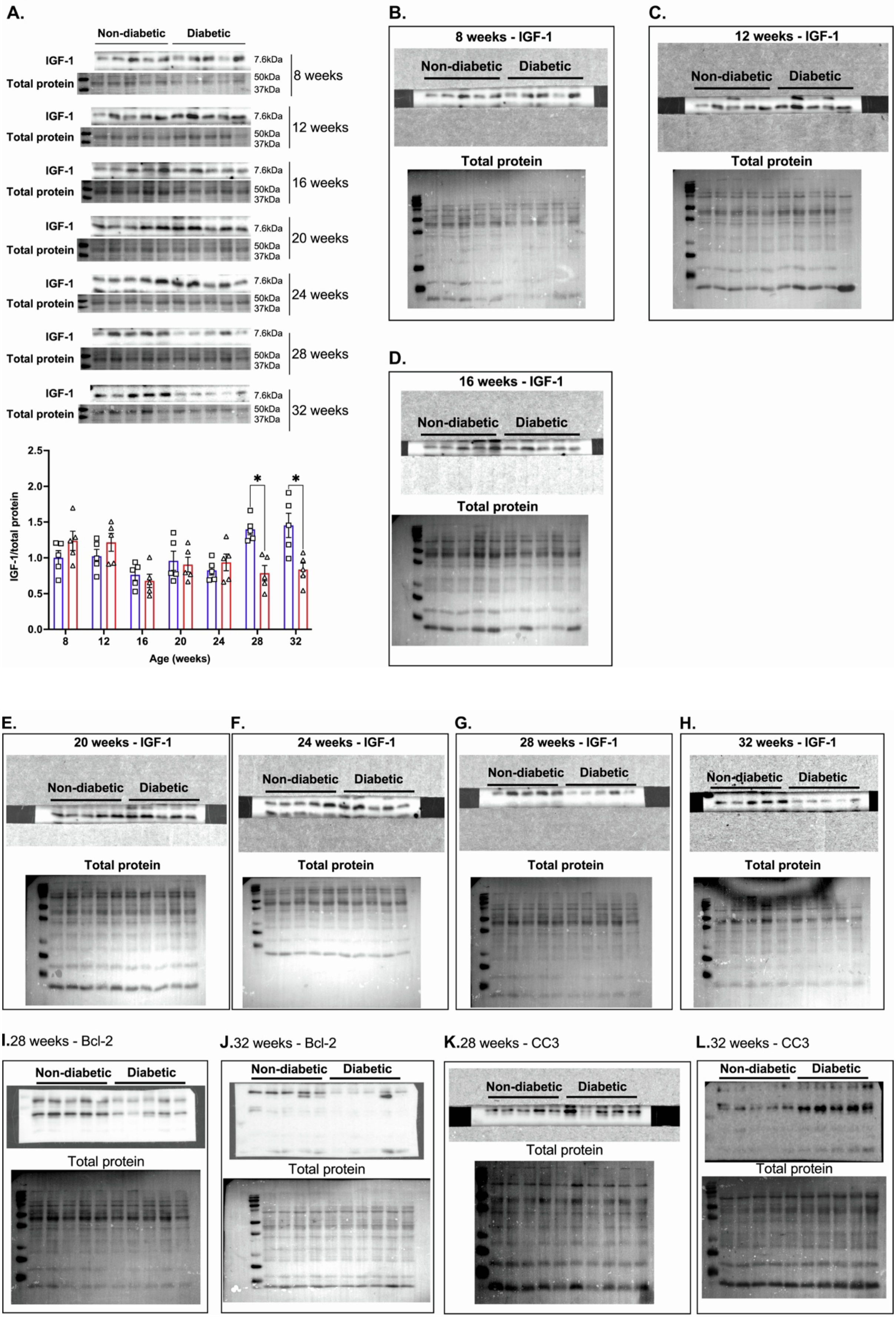

Supplemental Figure 2. A. Representative western blots and quantitative scatter plot bar graphs showing the expression of IGF-1 at different time points in the study groups. Data are represented as the ratio to total protein and are mean±SEM. Each western blot analysis was repeated three independent times. *P<0.05. **B-L.** Uncropped blots showing the expression of IGF-1 (**B-H, shown in Figure 2B and Supplemental Figure 2A**), Bcl-2 (**I&J, shown in Figure 2C**), and cleaved caspase-3 (CC3, **K&L, shown in Figure 2D**) in heart tissue collected from diabetic (db/db) and lean non-diabetic (db/+) mice.

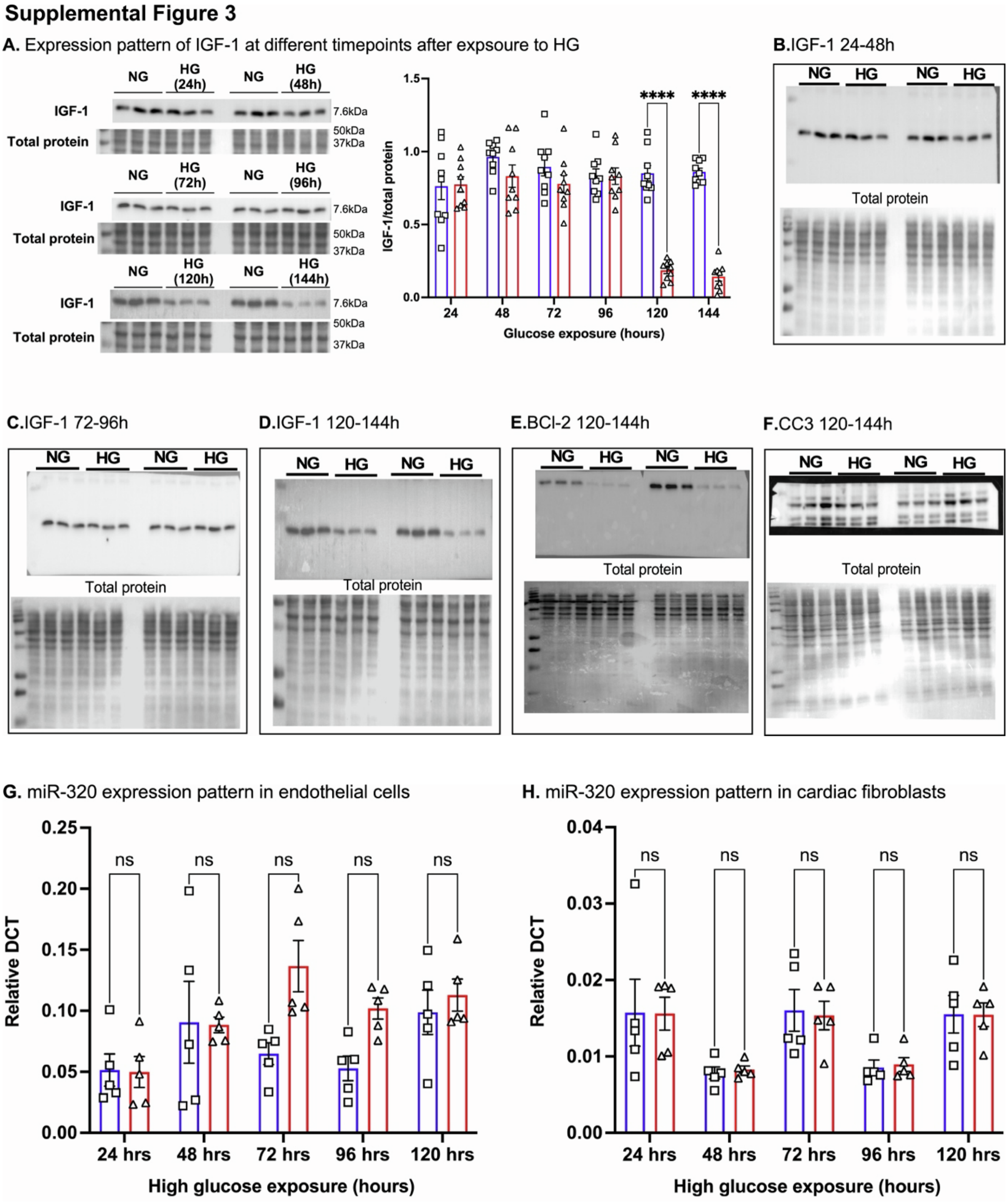

Supplemental Figure 3. A. Representative western blots and quantitative scatter plot bar graphs showing the expression of IGF-1 at different time points in the study groups. Data are represented as the ratio to total protein and are mean±SEM. Each western blot analysis was repeated three independent times. Uncropped blots showing the expression of IGF-1 (**B-D, shown in Figure 3B**), Bcl-2 (**E, shown in Figure 3C**), and cleaved caspase-3 (CC3, **F, shown**

in Figure 3D) in AC-16 human cardiomyocytes exposed to high glucose or mannitol (as osmotic control) for different time points. **G-H.** Quantitative scatter plot bar graphs showing miR-320 expression in the normal glucose (NG) and high glucose (HG) treated HUVEC (**G**) and cardiac fibroblasts (**H**) by RT-PCR analysis. Samples were collected at different points after exposing the cells to high glucose (HG) or mannitol for osmotic control (NG). Data are mean±SEM and expressed as relative DCT. n.s – not significant.

Supplemental Figure 4

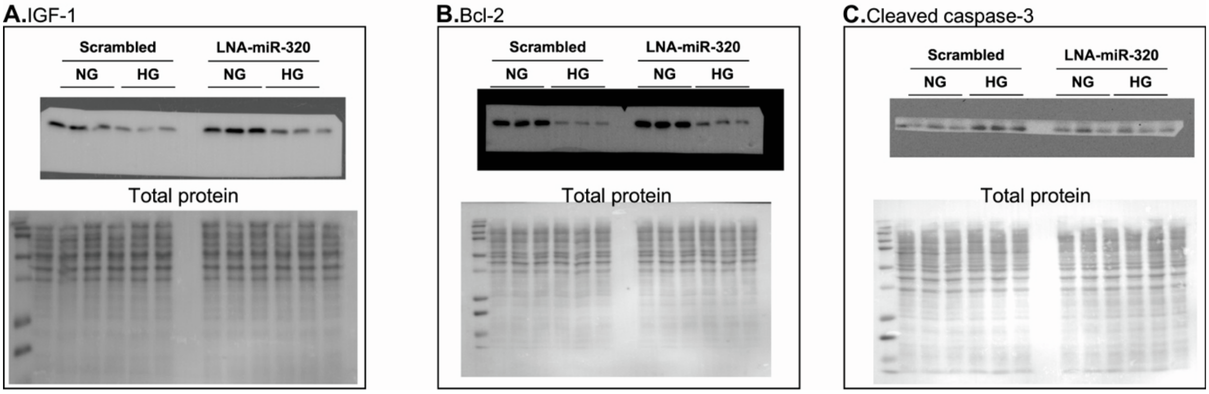

Supplemental Figure 4. Uncropped blots showing the expression of IGF-1 (**A, shown in Figure 4B**), Bcl-2 (**B, shown in Figure 4C**), and cleaved caspase-3 (CC3, **D, shown in Figure 4D**) in AC-16 human cardiomyocytes exposed to high glucose or mannitol (as osmotic control) and treated with either control scrambled sequence or LNA-miR-320 precursor to knockdown miR-320.

Supplemental Figure 5

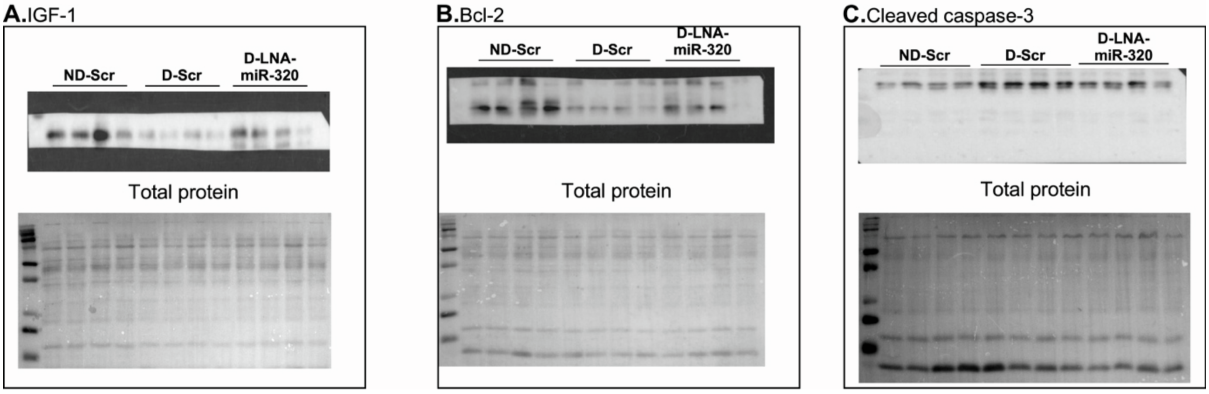

Supplemental Figure 5. Uncropped blots showing the expression of IGF-1 (**A, shown in Figure 5B**), Bcl-2 (**B, shown in Figure 5C**), and cleaved caspase-3 (CC3, **D, shown in Figure 5D**) in heart tissue collected from diabetic (db/db) and lean non-diabetic (db/+) mice following treatment with control scrambled sequence or LNA-miR-320 precursor to knockdown miR-320.

Supplemental Figure 6

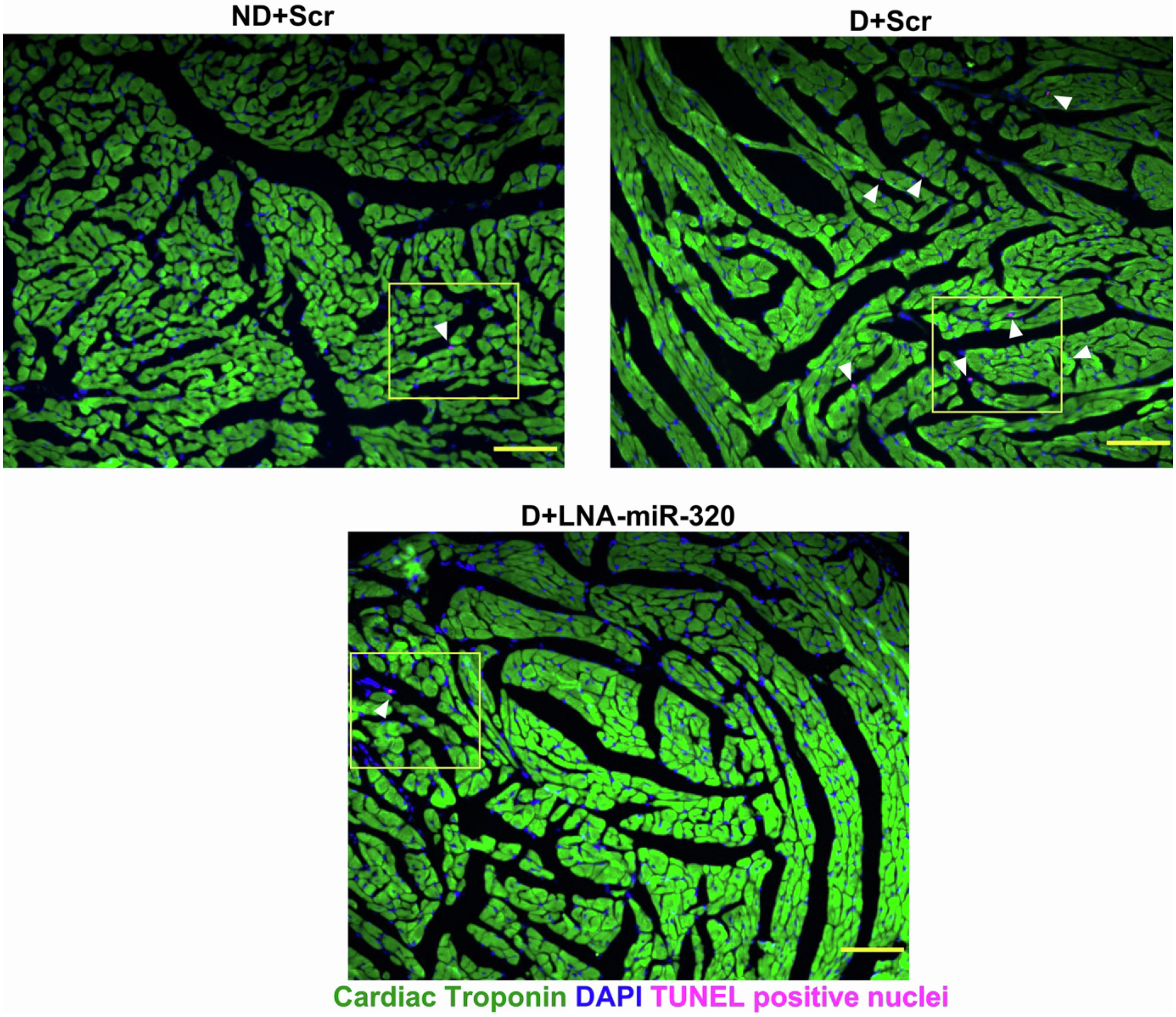

Supplemental Figure 6. Full fluorescent microscopy image of the section showing TUNEL positive (pink) cardiomyocytes (green) among the study groups. The white arrow points to the TUNEL positive cells. The yellow boxed area is zoomed in **Figure 5E**. Scale bars are 100 µm.

Supplemental Figure 7

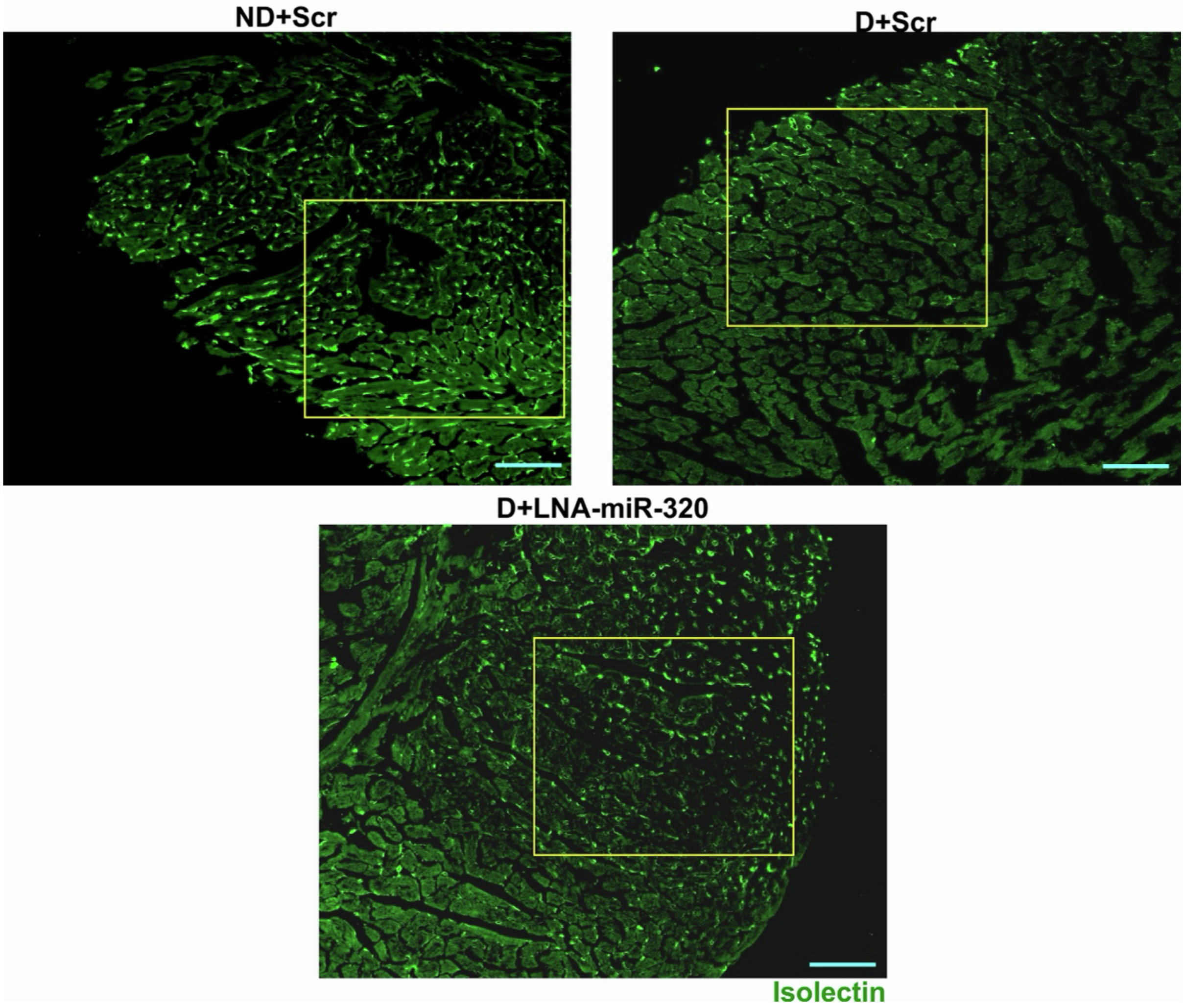

Supplemental Figure 7. Whole confocal microscopy of the section showing isolectin positive endothelial cells (green) among the study groups. The yellow boxed area is represented in **Figure 5F**. Scale bars are 100 μm.

Supplemental Figure 8

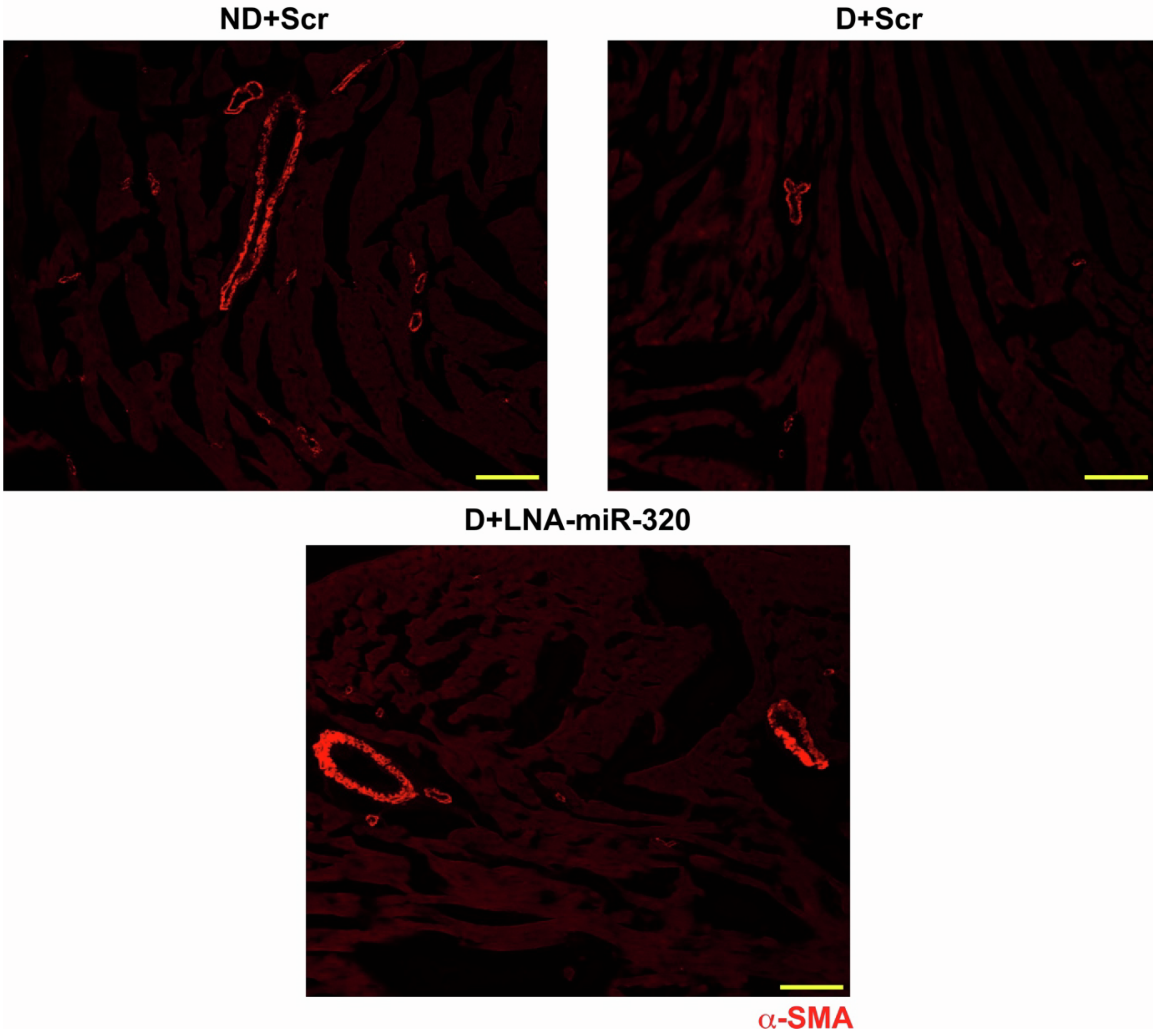

Supplemental Figure 8. Whole confocal microscopy of the section showing alpha-smooth muscle actin (α-SMA) positive cells (red) among the study groups. A smaller version of the image is represented in **Figure 5F**. Scale bars are 100 μm.
